# Antimicrobials: a global alliance for optimizing their rational use in intra-abdominal infections (AGORA)

**DOI:** 10.1186/s13017-016-0089-y

**Published:** 2016-07-15

**Authors:** Massimo Sartelli, Dieter G. Weber, Etienne Ruppé, Matteo Bassetti, Brian J. Wright, Luca Ansaloni, Fausto Catena, Federico Coccolini, Fikri M. Abu-Zidan, Raul Coimbra, Ernest E. Moore, Frederick A. Moore, Ronald V. Maier, Jan J. De Waele, Andrew W. Kirkpatrick, Ewen A. Griffiths, Christian Eckmann, Adrian J. Brink, John E. Mazuski, Addison K. May, Rob G. Sawyer, Dominik Mertz, Philippe Montravers, Anand Kumar, Jason A. Roberts, Jean-Louis Vincent, Richard R. Watkins, Warren Lowman, Brad Spellberg, Iain J. Abbott, Abdulrashid Kayode Adesunkanmi, Sara Al-Dahir, Majdi N. Al-Hasan, Ferdinando Agresta, Asma A. Althani, Shamshul Ansari, Rashid Ansumana, Goran Augustin, Miklosh Bala, Zsolt J. Balogh, Oussama Baraket, Aneel Bhangu, Marcelo A. Beltrán, Michael Bernhard, Walter L. Biffl, Marja A. Boermeester, Stephen M. Brecher, Jill R. Cherry-Bukowiec, Otmar R. Buyne, Miguel A. Cainzos, Kelly A. Cairns, Adrian Camacho-Ortiz, Sujith J. Chandy, Asri Che Jusoh, Alain Chichom-Mefire, Caroline Colijn, Francesco Corcione, Yunfeng Cui, Daniel Curcio, Samir Delibegovic, Zaza Demetrashvili, Belinda De Simone, Sameer Dhingra, José J. Diaz, Isidoro Di Carlo, Angel Dillip, Salomone Di Saverio, Michael P. Doyle, Gereltuya Dorj, Agron Dogjani, Hervé Dupont, Soumitra R. Eachempati, Mushira Abdulaziz Enani, Valery N. Egiev, Mutasim M. Elmangory, Paula Ferrada, Joseph R. Fitchett, Gustavo P. Fraga, Nathalie Guessennd, Helen Giamarellou, Wagih Ghnnam, George Gkiokas, Staphanie R. Goldberg, Carlos Augusto Gomes, Harumi Gomi, Manuel Guzmán-Blanco, Mainul Haque, Sonja Hansen, Andreas Hecker, Wolfgang R. Heizmann, Torsten Herzog, Adrien Montcho Hodonou, Suk-Kyung Hong, Reinhold Kafka-Ritsch, Lewis J. Kaplan, Garima Kapoor, Aleksandar Karamarkovic, Martin G. Kees, Jakub Kenig, Ronald Kiguba, Peter K. Kim, Yoram Kluger, Vladimir Khokha, Kaoru Koike, Kenneth Y. Y. Kok, Victory Kong, Matthew C. Knox, Kenji Inaba, Arda Isik, Katia Iskandar, Rao R. Ivatury, Maurizio Labbate, Francesco M. Labricciosa, Pierre-François Laterre, Rifat Latifi, Jae Gil Lee, Young Ran Lee, Marc Leone, Ari Leppaniemi, Yousheng Li, Stephen Y. Liang, Tonny Loho, Marc Maegele, Sydney Malama, Hany E. Marei, Ignacio Martin-Loeches, Sanjay Marwah, Amos Massele, Michael McFarlane, Renato Bessa Melo, Ionut Negoi, David P. Nicolau, Carl Erik Nord, Richard Ofori-Asenso, AbdelKarim H. Omari, Carlos A. Ordonez, Mouaqit Ouadii, Gerson Alves Pereira Júnior, Diego Piazza, Guntars Pupelis, Timothy Miles Rawson, Miran Rems, Sandro Rizoli, Claudio Rocha, Boris Sakakhushev, Miguel Sanchez-Garcia, Norio Sato, Helmut A. Segovia Lohse, Gabriele Sganga, Boonying Siribumrungwong, Vishal G. Shelat, Kjetil Soreide, Rodolfo Soto, Peep Talving, Jonathan V. Tilsed, Jean-Francois Timsit, Gabriel Trueba, Ngo Tat Trung, Jan Ulrych, Harry van Goor, Andras Vereczkei, Ravinder S. Vohra, Imtiaz Wani, Waldemar Uhl, Yonghong Xiao, Kuo-Ching Yuan, Sanoop K. Zachariah, Jean-Ralph Zahar, Tanya L. Zakrison, Antonio Corcione, Rita M. Melotti, Claudio Viscoli, Perluigi Viale

**Affiliations:** Department of Surgery, Macerata Hospital, Via Santa Lucia 2, 62100 Macerata, Italy; Department of Trauma Surgery, Royal Perth Hospital, Perth, Australia; Genomic Research Laboratory, Geneva University Hospitals, Geneva, Switzerland; Infectious Diseases Division, Santa Maria Misericordia University Hospital, Udine, Italy; Department of Emergency Medicine and Surgery, Stony Brook University School of Medicine, Stony Brook, NY USA; General Surgery Department, Papa Giovanni XXIII Hospital, Bergamo, Italy; Department of General, Maggiore Hospital, Parma, Italy; Department of Surgery, “Infermi” Hospital, Rimini, Italy; Department of Surgery, College of Medicine and Health Sciences, UAE University, Al-Ain, United Arab Emirates; Department of Surgery, UC San Diego Medical Center, San Diego, USA; Department of Surgery, University of Colorado, Denver Health Medical Center, Denver, CO USA; Department of Surgery, Division of Acute Care Surgery, and Center for Sepsis and Critical Illness Research, University of Florida College of Medicine, Gainesville, FL USA; Department of Surgery, University of Washington, Seattle, WA USA; Department of Critical Care Medicine, Ghent University Hospital, Ghent, Belgium; General, Acute Care, and Trauma Surgery, Foothills Medical Centre, Calgary, AB Canada; General and Upper GI Surgery, Queen Elizabeth Hospital, Birmingham, UK; Department of General, Visceral, and Thoracic Surgery, Klinikum Peine, Academic Hospital of Medical University Hannover, Peine, Germany; Department of Clinical microbiology, Ampath National Laboratory Services, Milpark Hospital, Johannesburg, South Africa; Department of Surgery, School of Medicine, Washington University in Saint Louis, Missouri, USA; Departments of Surgery and Anesthesiology, Division of Trauma and Surgical Critical Care, Vanderbilt University Medical Center, Nashville, TN USA; Department of Surgery, University of Virginia Health System, Charlottesville, VA USA; Departments of Medicine, Clinical Epidemiology and Biostatistics, and Pathology and Molecular Medicine, McMaster University, Hamilton, ON Canada; Département d’Anesthésie-Réanimation, CHU Bichat Claude-Bernard-HUPNVS, Assistance Publique-Hôpitaux de Paris, University Denis Diderot, Paris, France; Section of Critical Care Medicine and Section of Infectious Diseases, Department of Medicine, Medical Microbiology and Pharmacology/Therapeutics, University of Manitoba, Winnipeg, MB Canada; Australia Pharmacy Department, Royal Brisbane and Womens’ Hospital; Burns, Trauma, and Critical Care Research Centre, Australia School of Pharmacy, The University of Queensland, Brisbane, QLD Australia; Department of Intensive Care, Erasme Hospital, Université libre de Bruxelles, Brussels, Belgium; Department of Internal Medicine, Division of Infectious Diseases, Akron General Medical Center, Northeast Ohio Medical University, Akron, OH USA; Clinical Microbiology and Infectious Diseases, School of Pathology, Faculty of Health Sciences, University of the Witwatersrand, Johannesburg, South Africa; Division of Infectious Diseases, Los Angeles County-University of Southern California (USC) Medical Center, Keck School of Medicine at USC, Los Angeles, CA USA; Department of Infectious Diseases, Alfred Hospital, Melbourne, VIC Australia; Department of Surgery, College of Health Sciences, Obafemi Awolowo University, Ile-Ife, Nigeria; Division of Clinical and Administrative Sciences, College of Pharmacy, Xavier University of Louisiana, New Orleans, LA USA; Department of Medicine, Division of Infectious Diseases, University of South Carolina School of Medicine, Columbia, SC USA; General Surgery, ULSS19 del Veneto, Adria Hospital, RO, Italy; Biomedical Research Center, Qatar University, Doha, Qatar; Department of Microbiology, Chitwan Medical College, and Department of Environmental and Preventive Medicine, Oita University, Oita, Japan; Centre for Neglected Tropical Diseases, Liverpool School of Tropical Medicine, University of Liverpool, and Mercy Hospital Research Laboratory, Njala University, Bo, Sierra Leone; Department of Surgery, University Hospital Center, Zagreb, Croatia; Trauma and Acute Care Surgery Unit, Hadassah Hebrew University Medical Center, Jerusalem, Israel; Department of Traumatology, John Hunter Hospital and University of Newcastle, Newcastle, NSW Australia; Department of Surgery, Bizerte Hospital, Bizerte, Tunisia; Academic Department of Surgery, Queen Elizabeth Hospital, Birmingham, UK; Department of General Surgery, Hospital San Juan de Dios de La Serena, La Serena, Chile; Emergency Department, University of Leipzig, Leipzig, Germany; Department of Surgery, University of Colorado, Denver, CO USA; Department of Surgery, Academic Medical Centre, Amsterdam, The Netherlands; Department of Pathology and Laboratory Medicine, VA Boston HealthCare System, and Department of Pathology and Laboratory Medicine, Boston University School of Medicine, Boston, MA USA; Division of Acute Care Surgery, Department of Surgery, University of Michigan, Ann Arbor, MI USA; Department of Surgery, Radboud University Nijmegen Medical Center, Nijmegen, The Netherlands; Department of Surgery, Hospital Clínico Universitario, Santiago de Compostela, Spain; Pharmacy Department, Alfred Health, Melbourne, VIC Australia; Hospital Epidemiology and Infectious Diseases, Hospital Universitario Dr Jose Eleuterio Gonzalez, Monterrey, Mexico; Department of Pharmacology, Pushpagiri Institute of Medical Sciences and Research Centre, Thiruvalla, Kerala India; Department of General Surgery, Kuala Krai Hospital, Kuala Krai, Kelantan Malaysia; Department of Surgery and Obstetrics/Gynaecology, Regional Hospital, Limbe, Cameroon; Department of Mathematics, Imperial College London, London, UK; Department of Laparoscopic and Robotic Surgery, Colli-Monaldi Hospital, Naples, Italy; Department of Surgery, Tianjin Nankai Hospital, Nankai Clinical School of Medicine, Tianjin Medical University, Tianjin, China; Infectología Institucional SRL, Hospital Municipal Chivilcoy, Buenos Aires, Argentina; Department of Surgery, University Clinical Center of Tuzla, Tuzla, Bosnia and Herzegovina; Department General Surgery, Kipshidze Central University Hospital, Tbilisi, Georgia; Department of Surgery, Quatre Villes Hospital, St Cloud, France; School of Pharmacy, Faculty of Medical Sciences, The University of the West Indies, St. Augustine, Eric Williams Medical Sciences Complex, Uriah Butler Highway, Champ Fleurs, Trinidad and Tobago; Division of Acute Care Surgery, Program in Trauma, R Adams Cowley Shock Trauma Center, University of Maryland, Baltimore, MD USA; Department of Surgical Sciences, Cannizzaro Hospital, University of Catania, Catania, Italy; Ifakara Health Institute, Dar es Salaam, Tanzania; Department of Surgery, Maggiore Hospital, Bologna, Italy; Center for Food Safety, Department of Food Science and Technology, University of Georgia, Griffin, GA USA; School of Pharmacy and Biomedicine, Mongolian National University of Medical Sciences, Ulaanbaatar, Mongolia; Department of Surgery, University Hospital of Trauma, Tirana, Albania; Département d’Anesthésie-Réanimation, CHU Amiens-Picardie, and INSERM U1088, Université de Picardie Jules Verne, Amiens, France; Department of Surgery, Division of Burn, Critical Care, and Trauma Surgery (K.P.S., S.R.E.), Weill Cornell Medical College/New York-Presbyterian Hospital, New York, USA; Department of Medicine, Infectious Disease Division, King Fahad Medical City, Riyadh, Saudi Arabia; Department of Surgery, Pirogov Russian National Research Medical University, Moscow, Russian Federation; Sudan National Public Health Laboratory, Federal Ministry of Health, Khartoum, Sudan; Department of Surgery, Virginia Commonwealth University, Richmond, VA USA; Department of Global Health and Population, Harvard T.H. Chan School of Public Health, Boston, MA USA; Division of Trauma Surgery, Department of Surgery, School of Medical Sciences, University of Campinas (Unicamp), Campinas, SP Brazil; Institut Pasteur, Abidjan, Ivory Coast; 6th Department of Internal Medicine, Hygeia General Hospital, Athens, Greece; Department of General Surgery, Mansoura Faculty of Medicine, Mansoura University, Mansoura, Egypt; 2nd Department of Surgery, Aretaieion University Hospital, National and Kapodistrian University of Athens, Athens, Greece; Department of Surgery, Hospital Universitário Terezinha de Jesus, Faculdade de Ciências Médicas e da Saúde de Juiz de Fora, Juiz de Fora, Brazil; Center for Global Health, Mito Kyodo General Hospital, University of Tsukuba, Mito, Ibaraki Japan; Hospital Privado Centro Médico de Caracas and Hospital Vargas de Caracas, Caracas, Venezuela; Unit of Pharmacology, Faculty of Medicine and Defense Health, National Defence University of Malaysia, Kuala Lumpur, Malaysia; Institute of Hygiene, Charité-Universitätsmedizin Berlin, Hindenburgdamm 27, 12203 Berlin, Germany; Department of General and Thoracic Surgery, University Hospital Giessen, Giessen, Germany; Orgamed Consulting, Bad Griesbach, Germany; Department of Surgery, St. Josef Hospital, Ruhr University Bochum, Bochum, Germany; Department of Surgery, Faculté de médecine, Université de Parakou, BP 123 Parakou, Bénin; Division of Trauma and Surgical Critical Care, Department of Surgery, Asan Medical Center, University of Ulsan College of Medicine, Seoul, Republic of Korea; Department of Visceral, Transplant and Thoracic Surgery, Innsbruck Medical University, Innsbruck, Austria; Department of Surgery Philadelphia VA Medical Center, Perelman School of Medicine, University of Pennsylvania, Philadelphia, PA USA; Department of Microbiology, Gandhi Medical College, Bhopal, India; Clinic for Emergency Surgery, Medical Faculty University of Belgrade, Belgrade, Serbia; Department of Anesthesiology and Intensive Care, Charité Universitätsmedizin Berlin, Campus Benjamin Franklin, Berlin, Germany; 3rd Department of General Surgery, Jagiellonian University Medical College, Krakow, Poland; Department of Pharmacology and Therapeutics, College of Health Sciences, Makerere University, Kampala, Uganda; Department of Surgery, Albert Einstein College of Medicine and Jacobi Medical Center, Bronx, NY USA; Department of General Surgery, Division of Surgery, Rambam Health Care Campus, Haifa, Israel; Department of Emergency Surgery, City Hospital, Mozyr, Belarus; Department of Primary Care and Emergency Medicine, Kyoto University Graduate School of Medicine, Kyoto, Japan; Department of Surgery, The Brunei Cancer Centre, Jerudong Park, Brunei; Department of Surgery, Edendale Hospital, Pietermaritzburg, South Africa; School of Medicine, Western Sydney University, Campbelltown, NSW Australia; Division of Acute Care Surgery and Surgical Critical Care, Department of Surgery, Los Angeles County and University of Southern California Medical Center, University of Southern California, Los Angeles, CA USA; Department of General Surgery, Erzincan University, Faculty of Medicine, Erzincan, Turkey; Department of Pharmacy, Lebanese International University, Beirut, Lebanon; School of Life Science and The ithree Institute, University of Technology, Sydney, NSW Australia; Department of Biomedical Sciences and Public Health, Unit of Hygiene, Preventive Medicine and Public Health, UNIVMP, Ancona, Italy; Department of Critical Care Medicine, Cliniques Universitaires Saint Luc, Université Catholique de Louvain (UCL), Brussels, Belgium; Department of Surgery, Division of Trauma, University of Arizona, Tucson, AZ USA; Department of Surgery, Yonsei University College of Medicine, Seoul, South Korea; Texas Tech University Health Sciences Center School of Pharmacy, Abilene, TX USA; Department of Anaesthesiology and Critical Care, Hôpital Nord, Assistance Publique-Hôpitaux de Marseille, Aix Marseille Université, Marseille, France; Abdominal Center, University Hospital Meilahti, Helsinki, Finland; Department of Surgery, Inling Hospital, Nanjing University School of Medicine, Nanjing, China; Division of Infectious Diseases, Division of Emergency Medicine, Washington University School of Medicine, St. Louis, MO USA; Division of Infectious Diseases, Department of Clinical Pathology, Faculty of Medicine, University of Indonesia, Cipto Mangunkusumo General Hospital, Jakarta, Indonesia; Department for Traumatology and Orthopedic Surgery, Cologne Merheim Medical Center (CMMC), University of Witten/Herdecke (UW/H), Cologne, Germany; Health Research Program, Institute of Economic and Social Research, University of Zambia, Lusaka, Zambia; Multidisciplinary Intensive Care Research Organization (MICRO), Wellcome Trust-HRB Clinical Research, Department of Clinical Medicine, Trinity Centre for Health Sciences, St James’ University Hospital, Dublin, Ireland; Department of Surgery, Post-Graduate Institute of Medical Sciences, Rohtak, India; Department of Clinical Pharmacology, School of Medicine, University of Botswana, Gaborone, Botswana; Department of Surgery, Radiology, University Hospital of the West Indies, Kingston, Jamaica; General Surgery Department, Centro Hospitalar de São João, Porto, Portugal; Department of Surgery, Emergency Hospital of Bucharest, Bucharest, Romania; Center of Anti-Infective Research and Development, Hartford, CT USA; Department of Laboratory Medicine, Division of Clinical Microbiology, Karolinska University Hospital Huddinge, Stockholm, Sweden; Research Unit, Health Policy Consult, Weija-Accra, Ghana; Department of Surgery, King Abdullah University Hospital, Irbid, Jordan; Department of Surgery and Critical Care, Universidad del Valle, Fundación Valle del Lili, Cali, Colombia; Department of Surgery, Hassan II University Hospital, Medical School of Fez, Sidi Mohamed Benabdellah University, Fez, Morocco; Division of Emergency and Trauma Surgery, Ribeirão Preto Medical School, Ribeirão Preto, Brazil; Division of Surgery, Vittorio Emanuele Hospital, Catania, Italy; Department of General and Emergency Surgery, Riga East University Hospital ‘Gailezers’, Riga, Latvia; National Institute for Health Research, Health Protection Research Unit in Healthcare Associated Infections and Antimicrobial Resistance, Imperial College London, Hammersmith Campus, London, UK; Department of General Surgery, Jesenice General Hospital, Jesenice, Slovenia; Trauma and Acute Care Service, St Michael’s Hospital, University of Toronto, Toronto, Canada; U.S. Naval Medical Research Unit N° 6, Callao, Peru; General Surgery Department, Medical University, University Hospital St George, Plovdiv, Bulgaria; Intensive Care Department, Hospital Clínico San Carlos, Madrid, Spain; II Cátedra de Clínica Quirúrgica, Hospital de Clínicas, Universidad Nacional de Asunción, San Lorenzo, Paraguay; Department of Surgery, Catholic University of Sacred Heart, Policlinico A Gemelli, Rome, Italy; Department of Surgery, Faculty of Medicine, Thammasat University Hospital, Thammasat University, Pathum Thani, Thailand; Department of General Surgery, Tan Tock Seng Hospital, Tan Tock Seng, Singapore; Department of Gastrointestinal Surgery, Stavanger University Hospital, Stavanger, Department of Clinical Medicine, University of Bergen, Bergen, Norway; Department of Emergency Surgery and Critical Care, Centro Medico Imbanaco, Cali, Colombia; Department of Surgery, North Estonia Medical Center, Tallinn, Estonia; Surgery Health Care Group, Hull and East Yorkshire Hospitals NHS Trust, Hull, UK; APHP medical and infectious diseases ICU, Bichat Hospital, Paris, France; Institute of Microbiology, Biological and Environmental Sciences College, University San Francisco de Quito, Quito, Ecuador; Department of Molecular Biology, Tran Hung Dao Hospital, No 1, Tran Hung Dao Street, Hai Ba Trung Dist, Hanoi, Vietnam; 1st Department of Surgery - Department of Abdominal, Thoracic Surgery and Traumatology, General University Hospital, Prague, Czech Republic; Department of Surgery, Medical School University of Pécs, Pécs, Hungary; Nottingham Oesophago-Gastric Unit, Nottingham University Hospitals, Nottingham, UK; Department of Surgery, Sheri-Kashmir Institute of Medical Sciences, Srinagar, India; State Key Laboratory for Diagnosis and Treatment of Infectious Diseases, The First Affilliated Hospital, Zhejiang University, Zhejiang, China; Trauma and Emergency Surgery Department, Chang Gung Memorial Hospital, Taoyuan City, Taiwan; Department of Surgery, MOSC Medical College Kolenchery, Cochin, India; Infection Control Unit, Angers University, CHU d’Angers, Angers, France; Division of Trauma and Surgical Critical Care, DeWitt Daughtry Family Department of Surgry, University of Miami, Miami, FL USA; Anesthesia and Intensive Care Unit, AORN dei Colli Vincenzo Monaldi Hospital, Naples, Italy; Anesthesiology and Intensive Care Unit, Sant’Orsola University Hospital, Bologna, Italy; Infectious Diseases Unit, University of Genoa (DISSAL) and IRCCS San Martino-IST, Genoa, Italy; Infectious Diseases Unit, Department of Medical and Surgical Sciences, Sant’ Orsola Hospital, University of Bologna, Bologna, Italy

## Abstract

Intra-abdominal infections (IAI) are an important cause of morbidity and are frequently associated with poor prognosis, particularly in high-risk patients.

The cornerstones in the management of complicated IAIs are timely effective source control with appropriate antimicrobial therapy. Empiric antimicrobial therapy is important in the management of intra-abdominal infections and must be broad enough to cover all likely organisms because inappropriate initial antimicrobial therapy is associated with poor patient outcomes and the development of bacterial resistance.

The overuse of antimicrobials is widely accepted as a major driver of some emerging infections (such as *C. difficile*), the selection of resistant pathogens in individual patients, and for the continued development of antimicrobial resistance globally. The growing emergence of multi-drug resistant organisms and the limited development of new agents available to counteract them have caused an impending crisis with alarming implications, especially with regards to Gram-negative bacteria.

An international task force from 79 different countries has joined this project by sharing a document on the rational use of antimicrobials for patients with IAIs. The project has been termed AGORA (Antimicrobials: A Global Alliance for Optimizing their Rational Use in Intra-Abdominal Infections). The authors hope that AGORA, involving many of the world's leading experts, can actively raise awareness in health workers and can improve prescribing behavior in treating IAIs.

## Background

Judicious, careful and rational use of antimicrobials is an integral part of good clinical practice. This attitude maximizes the utility and therapeutic efficacy of treatment, and minimizes the risks associated with emerging infections and the selection of resistant pathogens. The indiscriminate and excess use of antimicrobial drugs appears the most significant factor in the emergence of resistant microorganisms in recent years.

We propose that clinical leaders drive antimicrobial stewardship and education programs to help standardize and improve prescribing behaviors. Furthermore, we argue that endorsement and guidance on the appropriate use of antimicrobials from leading scientific societies and clinical leaders within a specialty are vital to address the global threat of antimicrobial resistance and to provide support to policy makers.

AGORA, (Antimicrobials: A Global Alliance for Optimizing their Rational Use in Intra-Abdominal Infections) was conceived to actively raise the awareness of the rational and judicious use of antimicrobial medications in the treatment of intra-abdominal infections, in modern health care. This collaboration involves an international multidisciplinary task force, promoted by the World Society of Emergency Surgery (WSES), and endorsed by: the Surgical Infection Society (SIS), the American Association for the Surgery of Trauma (AAST), the Panamerican Trauma Society (PTS), the Indian Society for Trauma and Acute Care (ISTAC), the Korean Society of Acute Care Surgery (KSACS), the World Society of Abdominal Compartment Syndrome (WSACS), the South African Society of Clinical Microbiology (SASCM), the Hellenic Society for Chemotherapy, the Italian Society of Anti-Infective Therapy (SITA), The Italian Society of Anesthesiology, Analgesia, Resuscitation and Intensive Therapy (SIAARTI), the Italian Society of Surgery (SIC), the Italian Association of Hospital Surgeons (ACOI), the Italian Society of Emergency Surgery and Trauma (SICUT), the Italian Society of Intensive Care (SITI) and the World Alliance Against Antibiotic Resistance (WAAAR). WAAAR is a non-profit non-governmental organization participating actively in the global fight against antibiotic resistance.

It is the intent of AGORA to actively raise awareness of healthcare providers and improve prescribing behaviors when treating patients with IAIs worldwide.

This position paper aims to review the consequences of antimicrobial use, the evidence behind the global phenomenon of antimicrobial resistance, and to summarize the general principles of antimicrobial therapy in the modern management of patients with intra-abdominal infections. A review of the scientific rationale of modern antimicrobial pharmacotherapy is presented.

## Methods

An extensive review of the literature was conducted using the PubMed and MEDLINE databases, limited to the English language. The resulting information was shared by an international task force from 79 different countries combined in the AGORA (Antimicrobials: A Global Alliance for Optimizing their Rational Use in Intra-Abdominal Infections) project. The resulting document, detailing current knowledge and opinion, is presented in this position and consensus statement. The document is presented in light of the aim to facilitate clinical guidance in the rational use of antimicrobials for intra-abdominal infections.

## Results

### The development of antimicrobial resistance and the selection of pathogenic bacteria from use of antibiotics

Clinicians prescribing antibiotics have two potentially conflicting responsibilities. First, clinicians should offer optimal therapy for the individual patient under their care by offering antimicrobials. Second, they should preserve the efficacy of antimicrobials and minimize the development of resistance and the selection of resistant pathogens [1] by withholding antimicrobials.

#### Antimicrobials and resistance

*The problem of antimicrobial resistance (AMR) is widespread worldwide. Clinicians should be aware of their role and responsibility for maintaining the effectiveness of current and future antimicrobials. Health workers can help tackle resistance by:*

- *enhancing infection prevention and control;*
- *prescribing and dispensing antimicrobials when they are truly needed; and*
- *prescribing and dispensing the right antimicrobial(s) to treat the illness.*

Infections caused by antibiotic-resistant bacteria continue to be a challenge. Rice [2] in 2008 coined the acronym of “ESKAPE” pathogens including *Enterococcus faecium, Staphylococcus aureus, Klebsiella pneumoniae, Acinetobacter baumannii, Pseudomonas aeruginosa,* and *Enterobacter* species to emphasize that these bacteria currently cause the majority of hospital infections and effectively “escape” the effects of antibacterial drugs [3].

Although the phenomenon of AMR can be attributed to many factors, there is a well-established relationship between antimicrobial prescribing practices and the emergence of antimicrobial resistant pathogens [4–6]. After they have emerged, resistant pathogens may be transmitted from one individual to another [7]. While, the indigenous intestinal microbiota provides an important host-defense mechanism by preventing colonization of potentially pathogenic microorganisms, the intestinal tract is also an important reservoir for antibiotic-resistant bacteria [8, 9]. Antibiotics exert undue selective pressure on bacteria in the intestine through a two-step process. First, antibiotics kill susceptible bacteria from the commensal intestinal microbiota. This favors bacteria within the intestine that are already resistant, have become resistant through mutation or through the acquisition of exogenous DNA (e.g. plasmids) from cells colonized in, or passing through, the intestinal tract. Most feared by clinicians is the acquisition of multi-drug resistant organisms (MDRO) in the intestinal microbiota of patients [10]. Second, antibiotics promote the overgrowth of MDRO present in the intestinal microbiota [11, 12] thereby increasing the risks of cross-transmission between patients [13] and increasing the risk of untreatable or difficult-to-treat infectious outbreaks [14]. Selective pressure from antibiotics combined with ineffective infection control practices accelerates the spread of resistant bacteria [15]. Thus, with few new antibiotics being developed, particularly for Gram-negative organisms, prudent antibiotic use is vital for delaying the emergence of resistance [16].

#### Antibiotics and *C. difficile* infection

*C. difficile infections have become more frequent, more severe and more difficult to treat.*

*The prolonged use of antibiotics induces a change in the intestinal flora and may result in a higher incidence of C. difficile infections.*

*C. difficile* is an anaerobic, spore forming, Gram-positive bacillus, which may be part of the normal intestinal microbiota in healthy newborns but is rarely present in the gut of healthy adults [17]. A direct correlation between antibiotic use and *C. difficile* infection (CDI) has been well described [18]. Disruption of the normal gut flora as a consequence of antibiotic use provides an excellent setting for *C. difficile* to proliferate and produce toxins [19].

The risk of CDI is increased up to 6-fold during and in the subsequent month after antibiotic therapy [20]. Although nearly all antibiotics have been associated with CDI, clindamycin, amoxicillin-clavulanate, cephalosporins and fluoroquinolones have traditionally been considered to pose the greatest risk [21–38].

In 2014, a systematic review of observational epidemiological studies measuring associations between antibiotic classes and hospital acquired CDI was published [30]. Of 569 citations identified, 13 case–control and 1 cohort study (15,938 patients) were included. The strongest associations were found for third-generation cephalosporins, clindamycin, second-generation cephalosporins, fourth-generation cephalosporins, carbapenems, trimethoprim-sulphonamides, fluoroquinolones and penicillin combinations.

In the last two decades, the dramatic increase in incidence and severity of CDI in many countries worldwide [18], has made CDI a global public health challenge.

CDI represents the most common cause of diarrhea in hospitalized patients. *C. difficile* colitis can be treated by oral or intravenous metronidazole and/or oral or intracolonic vancomycin [29]. In severe colitis, surgery may be required [30].

In 2015, the WSES published guidelines for management of *C. difficile* infection in surgical patients [18].

#### Antibiotics and invasive candidiasis

*Usually, Candida spp. are kept under control by the native bacteria and by the body's immune defenses. Antibiotics disrupt normal bacterial colonization and may create an environment in which fungi can thrive.*

The gastrointestinal tract is normally colonized by yeasts, mainly *Candida spp.* [31–33]. It is believed that invasive candidiasis predominantly originates from this reservoir [34]. The mechanisms that allow *Candida spp.* to cause invasive candidiasis and candidemia are quite complex. *Candida spp.* are commensal members of the gastrointestinal microflora and in homeostasis with the host. However, when this homeostasis is disrupted, the yeast can break through the intestinal mucosal barrier and cause dissemination [35, 36]. This process may involve many contributing factors and multiple mechanisms [35]. The immunocompetent human host, with his/her resident microbiota, is remarkably good at maintaining a healthy symbiotic equilibrium. However, chemotherapy and invasive surgical procedures, may result in a human host disequilibrium that facilitate fungal invasion.

#### The global burden of antimicrobial resistance

*Antimicrobial Resistance (AMR) poses a global challenge. No single country, however effective it is at containing resistance within its boundaries, can protect itself from the importation of MDRO through travel and trade.*

*The global nature of AMR calls for a global response, both in the geographic sense and across the whole range of sectors involved. Nobody is exempt from the problem, nor from playing a role in the solution.*

*Despite an increasing prevalence of MDRO worldwide, the health and economic impact of these organisms is often underestimated.*

*The impact of AMR worldwide is significant, both in economic terms, and clinical morbidity and mortality because it may:*

- *lead to some infections becoming untreatable;*
- *lead to inappropriate empirical treatment in critically ill patients where an appropriate and prompt treatment is mandatory;*
- *increase length of hospital stay, morbidity, mortality and cost; and*
- *make necessary alternative antimicrobials which are more toxic, less effective, or more expensive.*

Antimicrobial resistance is a natural phenomenon that occurs as microbes evolve. However, human activities have accelerated the pace at which microorganisms develop and disseminate resistance. Incorrect and injudicious use of antibiotics and other antimicrobials, as well as poor prevention and control of infections, are contributing to the development of such resistance.

The impact of AMR worldwide is significant, both in economic terms, and clinical morbidity and mortality [38–40].

Although the optimally effective and cost-effective strategy to reduce AMR is not known, a multifaceted approach is most likely to be successful [15].

Many calls to action on antimicrobial resistance have been made over the past years, but there has been very little progress. Countries with the strictest policies on antibiotic prescription including Scandinavian countries the Netherlands and Switzerland now report the lowest rates of bacterial resistance [41]. However in most high income countries, clinical use of antibiotics has not declined, despite frequent calls to curtail overuse.

The World Health Organization (WHO) is now leading a global effort to address antimicrobial resistance. At the 68th World Health Assembly in May 2015, the World Health Assembly endorsed a global action plan to tackle antimicrobial resistance [42]. It sets out five strategic objectives:

- to improve awareness and understanding of antimicrobial resistance;
- to strengthen knowledge through surveillance and research;
- to reduce the incidence of infection;
- to optimize the use of antimicrobial agents; and
- to develop the economic case for sustainable investment that takes account of the needs of all countries, and increase investment in new medicines, diagnostic tools, vaccines and other interventions.

An alarming pattern of resistance involving multi and pandrug-resistant Gram-negative bacteria is currently emerging; multi-resistant *Enterobacteriaceae* is an increasing major concern worldwide [43, 44]. Comparative antimicrobial resistance data worldwide are difficult to obtain and inevitably suffer from bias. In high income countries, MDRO have historically been confined to the hospital setting. Since the middle of the 2000s, however, MDRO such as the extended-spectrum (ESBL) producing beta-lactamase (ESBL) *Enterobacteriaceae* have been widespread in the community setting [45].

Throughout the 1980s and 1990s, prolonged hospital and intensive care unit stays were considered among the most important risk factors for harboring ESBL *Enterobacteriaceae* along with exposure to broad-spectrum antibiotics [46].

However, most ESBL producing infections are now also in the community and healthcare-associated settings as demonstrated in studies from Europe and the Southeastern USA [47, 48].

The burden of MDRO infections in low-middle income countries (LMIC) is difficult to quantify, because surveillance activities to guide interventions require resources [49].

In these countries, routine microbiologic culture and sensitivity testing, especially in rural hospitals, are not performed, due to lack of personnel, equipment and financial resources. As a result antimicrobial therapy is empirical and a small collection of antimicrobials may be overused. This approach, although relatively inexpensive, may further increase the emergence of AMR and hence sub-optimal clinical outcomes [49].

Therefore, although resistance containment interventions in healthcare structures have mostly been implemented in high-income countries, there is a pressing need to intervene in the resistance pandemic also in LMIC.

### Mechanism of resistance

*The treatment of infections is increasingly complicated by the ability of bacteria to develop resistance to antibiotics.*

*Bacteria may be intrinsically resistant to one or more classes of antibiotics, or may acquire resistance by de novo mutation or by the acquisition of resistance genes from other organisms.*

*Better understanding of mechanisms of antibiotic resistance would allow the development of control strategies to reduce the spread of resistant bacteria and their evolution.*

Bacteria may be intrinsically resistant to a class of antibiotics or may acquire resistance.

Main mechanisms of resistance to antibiotics can be caused by [50]:

- the inactivation or modification of the antibiotic;
- an alteration or the protection of the target site of the antibiotic that reduces its binding capacity;
- the modification of metabolic pathways to circumvent the antibiotic effect; and
- the reduced intracellular antibiotic accumulation by decreasing permeability and/or increasing active efflux of the antibiotic.

Bacteria can develop resistance to antibiotics by mutating existing genes (vertical evolution) [51], or by acquiring new genes from other strains or species (horizontal gene transfer) [52].

Many of the antibiotic resistance genes are carried on genetic elements (plasmids, transposons or phages) that act as vectors that transfer these genes to other members of the same bacterial species, as well as to bacteria in another genus or species [52].

#### Evolution and dissemination of resistance

One of the main problems surrounding antibiotic resistance genes is their association with mobile genetic elements (MGEs) such as conjugative plasmids, transposons, and viruses or mobility genes from MGEs [53–55]. The MGEs allow resistance to spread horizontally and disseminate among different bacterial species. Although this association seems improbable, it appears to occur frequently and follows a series of evolutionary steps fueled by natural selection (antibiotic selection). The power of modern DNA sequence analysis allows us to better understand the process of emergence of these genetic structures.

Most families of antibiotics present in nature are compounds produced by fungi or bacteria; bacteria utilize these compounds to eliminate competitor microorganisms. As part of this arms race, many microorganisms code for genes whose products neutralize antibiotics; these genes may have been present in bacterial chromosomes for millions of years and they were probably not mobile, as evidenced by recent findings. The massive use of antibiotics probably favored selection of antibiotic resistant bacteria resulting in large numbers of bacteria coding for resistance genes. Additionally, genes with mutations conferring novel forms of antibiotic resistance may also rise in numbers under antibiotic pressure.

On the other hand, bacterial chromosomes are populated with transposable elements (insertion sequences known as *ISs*), which jump frequently and randomly, as demonstrated during in vitro experiments [56]. The existence of large numbers of bacteria containing resistance genes sets the stage for the next step which is the association of AR genes with *IS,* which may cause increased transcription of resistance genes (*IS* contain powerful promoters) [57]; antibiotic selection once again will then favor the survival of bacteria with higher expression of resistance genes. *IS*s are also known to promote the mobilization of contiguous pieces of DNA and once there is a large number of bacteria with resistance genes associated to *IS*s, the stage is set for the next step which is the mobilization of the resistance genes.

Antibiotic resistance genes could be mobilized to genetic structures, such as plasmids and phages, which can move horizontally between bacterial cells including different bacterial species. This is probably the path followed by many plasmid encoded genes such as CTX-M-type β-lactamase (found in plasmids in *Enterobacteriaceae*), which was probably mobilized by a transposon from its original location in the chromosome of the intestinal bacteria *Kluyvera* [58].

The association of resistance genes to these mobile structures could occur through *IS*s (as explained previously); this has been postulated as the origin of many MGE. Alternatively, plasmids or phages may also integrate in the bacterial chromosome in the vicinity of resistance genes and then mobilize the resistance genes as these structures excise from chromosomes [55].

Some of these gene associations are ancient and they have been dragging genes that confer bacteria with different abilities including protection from harmful compounds. Some of them have lost most of the genetic information retaining few MGE genes such as transposases, integrases or genes involved in conjugation (relaxosome) [55]. The examples of these ancient platforms are integrons which have integrases derived from phages [55] and conjugative integrative elements such as the staphylococcal SCC*mec* which contains genes allowing conjugation (similar to plasmids) [59]. Additionally, conjugal plasmid integration (formation of Hfr) and phages (generalized transduction) could promote the transfer of large sections of bacterial genome including resistance genes) [60, 61].

The antibiotic selection is responsible for large numbers of bacteria with antibiotic resistance genes; the combination of a large number of resistance genes and recombinant nature of bacterial chromosomes creates the ideal scenario for combination of genes. Antibiotic selection will influence every single genetic event (recombination, excision, conjugation, integration) allowing the survival of bacteria with ideal resistance gene expression; only changes that are meaningful (high benefits and low cost) will prevail, the rest of them will disappear or they will circulate at undetectable levels. The MGEs detected by the current gene detection analysis, including metagenomic analysis, correspond to the tip of the iceberg, as they represent the most successful gene associations. The more we use antibiotics, the more efficient MGEs will evolve.

#### Antibiotic resistance in *Enterobacteriaceae*

##### Resistance to beta-lactam antibiotics

Resistance to beta-lactams in *Enterobacteriaceae* is mainly conferred by beta-lactamases. These enzymes inactivate beta-lactam antibiotics by hydrolysis. Beta-lactamases are commonly classified according to two systems: the Ambler molecular classification and the Bush–Jacoby–Medeiros functional classification [62].

The Ambler scheme classifies beta-lactamases into four classes according to the protein homology of enzymes. Beta-lactamases of class A, C, and D are serine beta-lactamase and class B enzymes are metallo-beta-lactamases [63]. The Bush–Jacoby–Medeiros functional scheme is based on functional properties of enzymes and on their ability to hydrolyze specific beta-lactam classes [64]. This classification was updated in 2010 [65]. The updated system includes group 1 (class C) cephalosporinases; group 2 (classes A and D) broad-spectrum, inhibitor-resistant, extended-spectrum beta-lactamases and serine carbapenemases; and group 3 (class B) metallo-beta-lactamases [65].

Group 1 enzymes are cephalosporinases belonging to molecular class C. They are more active on cephalosporins than benzylpenicillin. It includes AmpC beta-lactamases. AmpC beta-lactamases are clinically important cephalosporinases capable of inactivating cephalotin, cefazolin, cefoxitin, most penicillins, and beta-lactamase inhibitor-beta-lactam combinations. AmpC-hyperproducing mutants are resistant to penicillins, aztreonam, third generation cephalosporins including cefotaxime, ceftazidime, ceftriaxone and even ertapenem when the enzyme is massively expressed. Imipenem, meropenem and doripenem remain the most active beta-lactams against AmpC beta-lactamases [66].

Cefepime, a fourth-generation cephalosporin with broader spectrum activity compared to ceftriaxone, is a poor inducer of AmpC beta-lactamase. Many AmpC-producing organisms are susceptible to cefepime because cefepime is poorly hydrolyzed by the AmpC beta-lactamase enzyme [67]. However, the role of cefepime in treating infections caused by AmpC-producing organisms is controversial because of the inoculum effect. In vitro studies showed a high inoculum effect. If a 100-fold-higher inoculum is used, cefepime MIC increases dramatically for some AmpC producers [68]. Clinical studies demonstrated that cefepime may be a reasonable option for the treatment of invasive infections due to AmpC beta-lactamase-producing organisms when adequate source control is achieved [69].

Group 2 (classes A and D) represent the largest group of beta-lactamases, it includes ESBL producing *Enterobacteriaceae* and carbapenemases (class A) and OXA beta-lactamases (class D).

ESBL are enzymes capable of hydrolyzing and inactivating a wide variety of beta-lactams, including third-generation cephalosporins, penicillins, and aztreonam [70–72]. Most ESBLs of clinical interest are encoded by genes located on plasmids. These plasmids may also carry genes encoding resistance to other multiple drug classes including aminoglycosides and fluoroquinolones [73].

The main ESBL enzymes imparting antibiotic resistance are TEM-, SHV-, and CTX-M.

Although hyperproduction of beta-lactamases or additional resistance mechanisms may hamper the antibiotic effectiveness, most TEM, SHV and CTX-M variants remain susceptible in vitro to beta-lactam/beta-lactam inhibitor combinations (BLBLI) such as amoxicillin-clavulanate or piperacillin-tazobactam. However, the efficacy of BLBLI for treating serious ESBL infections is controversial [66]. For example, the increase in piperacillin/tazobactam MICs when bacterial inocula reach 10^7^ colony forming units/mL is concerning, and may indicate the presence of other mechanisms of resistance [73]. Furthermore, in critically ill patients the pharmacokinetic properties of beta-lactams are modified and these patients may have adverse outcomes as a result of sub-optimal antibiotic exposure [74].

Rates of CTX-M infections have increased during the last decade compared with rates of TEM- and SHV- infections [75]. The diffusion of CTX-M-producing *Enterobacteriaceae* are common in Southeast Asia and Eastern Mediterranean countries (estimated rates of intestinal carriage, ~60 and ~30 %, respectively), therefore travel in these areas is a risk factor for acquisition [66, 76]. Carriage rates in the community are now above 5–10 % in many other geographic areas [76].

The OXA-type beta-lactamases are so named because of their oxacillin-hydrolyzing abilities. OXA beta-lactamases have resistance limited to the penicillins, but some became able to confer resistance to cephalosporins [77, 78]. OXA-1 and OXA-10 beta-lactamases have only a narrow hydrolytic spectrum. However, other OXA beta-lactamases including OXA-11, -14, -15, -16, -28, -31, -35 and -45 confer resistance to cefotaxime, ceftazidime and aztreonam [66]. OXA-23 and OXA-48 are classes of carbapenemases that belong to OXA-type beta-lactamases with carbapenem-hydrolyzing activities [79, 80]. While OXA-23 appears most frequently in *A. baumannii*, OXA-48 enzymes have now become widespread in the *Enterobacteriaceae,* especially in Mediterreanean countries [79].

*K. pneumoniae* carbapenemases (KPCs) are beta-lactamases produced by Gram-negative bacteria. They efficiently hydrolyse penicillins, all cephalosporins, monobactams, beta-lactamase inhibitors, and even carbapenems. KPCs are becoming an increasingly significant problem worldwide [81]. The first plasmid-encoded serine carbapenemase in the KPC enzyme family was discovered in the USA in 1996 and reported in 2001 [66]. KPC is the most common carbapenemase in the United States and in some European countries such as Italy. However, different groups of enzymes possessing carbapenemase properties have emerged, and are spreading worldwide. KPC-producing *K. pneumoniae* pose a serious threat in clinical situations where administration of effective empiric antibiotics is essential to prevent mortality following bacteraemia and infections in immunocompromised patients including organ transplant recipients and those with cancer [81–86]. A major concern is the emergence of colistin resistant KPC-positive *K pneumoniae* isolates. This is of particular clinical relevance, as colistin is currently a key component of treatment combinations. The selection of colistin resistant KPC producing strains probably results from the increasing use of colistin, in areas where KPC-positive *K pneumoniae* have spread [87]. In light of the emergence of a plasmid-borne colistin resistance gene, the prudent use of colistin is warranted [43].

Group 3 (Class B) metallo-beta-lactamases (MBLs) differ structurally from the other beta-lactamases by their requirement for a zinc ion at the active site. They are all capable of hydrolysing carbapenems. In contrast to the serine beta-lactamases, the MBLs have poor affinity or hydrolytic capability for monobactams and are not inhibited by clavulanic acid or tazobactam. The most common metallo-beta-lactamase families include the IMP, VIM and NDM. [88, 89]. A currently emerging MBL is the NDM (New Delhi metallo-beta-lactamase). NDM-1 was first detected in 2008 in a patient returning to Sweden from India [90, 91]. NDM-1 has been shown to be present at significant frequency within *Enterobacteriaceae* in India and has subsequently been shown to be present in bacterial isolates in a number of countries worldwide [91].

##### Resistance to fluoroquinolones

All *Enterobacteriaceae* are naturally susceptible to fluoroquinolones. The process by which susceptible strains become highly fluoroquinolone resistant is thought to be a result of a series of sequential steps and several mutations are needed to produce a high level of fluoroquinolone resistance [92, 93]. High-level resistance emerges after successive chromosomal mutations in the DNA gyrase- encoding *gyrA* gene and topoisomerase IV-encoding *parC* gene [93]. The over-expression of efflux pumps may also play a role in the high level of resistance in certain strains. While there are many genes that are assumed to encode a drug transporter protein in *Enterobacteriaceae*, only AcrAB/TolC overexpression plays a major role in *E. coli* as a main efflux pump implicated in extruding fluoroquinolones [92]. Resistance to fluoroquinolones can also be mediated by the plasmid-encoded *qnr* genes, which confer protection of bacterial topoisomerases against fluoroquinolones, the plasmid-encoded efflux pump *qepA* and the aminoglycoside-modifying enzyme AAC(6′)-Ib-cr that partly inactivate ciprofloxacin [92].

##### Resistance to aminoglycosides

Aminoglycosides resistance occurs through several mechanisms that can simultaneously coexist. Aminoglycosides resistance in *Enterobacteriaceae* relies mainly on the genes encoding aminoglycoside-modifying enzymes (AMEs). AMEs hamper antibiotic activity. AMEs are often located on plasmids that carry multiple resistance genes, including ESBL [66]. Current rates of co-resistance in hospital-acquired ESBL are 50–60 % for gentamicin and 10–20 % to amikacin [66]. Other described mechanisms of resistance include modification of the antibiotic target by mutation of the 16S rRNA or ribosomal proteins, methylation of 16S rRNA (by RNA methlysases which genes are often co-located with beta-lactamase encoding genes), reduced permeability and/or increasing active efflux of the antibiotic [94].

#### Antibiotic resistance in Non-fermenting gram-negative bacteria

Non-fermenting Gram-negative bacteria (*P. aeruginosa, S. maltophilia* and *A. baumannii*) are intrinsically resistant to many drugs and can acquire resistance to virtually any antimicrobial agent. A variety of resistance mechanisms have been identified in *P. aeruginosa* and other Gram-negative non-fermenting bacteria, including impermeable outer membranes, expression of efflux pumps, target alteration and production of antibiotic-hydrolyzing enzymes such as AmpC beta-lactamases that are either chromosomally encoded or acquired [95]. These mechanisms may be present simultaneously, conferring multiresistance to different classes of antibiotics. These mechanisms may also allow transmission to multiple strains of bacteria [66].

*P. aeruginosa* is intrinsically resistant to a number of beta-lactam antibiotics including amoxicillin, first and second generation cephalosporins, cefotaxime, ceftriaxone and ertapenem. Effective agents include ticarcillin, piperacillin, ceftazidime, cefepime, imipenem, meropenem and doripenem. Aztreonam activity is variable [66]. Unlike tazobactam, clavulanate is a strong inducer of AmpC in *P. aeruginosa* [96] *P. aeruginosa* also has the ability to acquire beta-lactamases, including ESBL and carbapenemases [66]. The *P. aeruginosa* genome contains several different multidrug resistance efflux pumps, which reside in the membrane and remove antimicrobials and toxins, thereby lowering their concentration inside the cell to sub-toxic levels. Overproduction of these pumps reduces susceptibility to a variety of antibiotics [97]. The most common system is MexAB-OprM. Its overexpression confers resistance to ticarcillin, aztreonam, and at a lesser extent, meropenem [98]. Reduced outer-membrane permeability caused by qualitative or quantitative alterations of the OprD porin, which manages the passage of imipenem through the outer membrane, confers *P. aeruginosa* a basal level of resistance to carbapenems, especially to imipenem [99].

The mechanisms of AMR in *A. baumannii* are various, and generally include production of beta-lactamases, impermeable outer membrane, expression of efflux pumps, and change of targets or cellular functions such as alterations in penicillin-binding proteins (PBPs). The PBPs play a crucial role in the synthesis of peptidoglycan, an essential component of the bacterial cell wall. *A. baumannii* naturally produces a non-inducible AmpC-type cephalosporinase (ACE-1 or ACE-2) and an OXA-51-like carbapenemase which confers, at basal levels of expression, intrinsic resistance to aminopenicillins, first and second generation cephalosporins and aztreonam. Ertapenem naturally lacks activity against non-fermenting Gram negative bacteria including *A. baumannii* [100]. Overproduction of the AmpC-type cephalosporinase confers acquired resistance to carboxypenicillins, ureidopenicillins and third generation cephalosporins. The emergence of carbapenem-resistant clones of *A baumannii* has been reported since the late 1980s. Carbapenem resistance can result from the over-expression of OXA-51-like oxacillinase, and from the acquisition of OXA-23-like, IMP, VIM, SIM or, more recently, NDM-type carbapenemases [101]. Acquired resistances to fluoroquinolones (mutations in *gyrA* and/or *parC*) and aminoglycosides (plasmid-borne AMEs) may be observed in ESBL as well as carbapenemase-producing *A. baumannii* strains [66]. Colistin resistant isolates are now increasing worldwide. Resistance to colistin is thought to be mediated by modifications of the lipopolysaccharides of the bacterial cell membrane that interfere with the agent’s ability to bind bacterial targets [100].

#### Antibiotic resistance in Enterococci

Enterococci are intrinsically resistant to some penicillins, all cephalosporins, and, at a low level, to aminoglycosides. Additionally, they have acquired resistance to many other classes of antibiotics [102, 103].

Enterococci have intrinsic resistance to most beta-lactam antibiotics because of the low affinity penicillin binding proteins (PBPs). Attachment of beta-lactam agents to PBPs results in impaired cell wall synthesis and, in most cases, programmed cell death via creation of reactive oxygen species. Enterococci express low-affinity PBPs (PBP5 in *E. faecium*, PBP4 in *E. faecalis*) that bind weakly to beta-lactam antibiotics. Enterococci may develop increased resistance to penicillins through acquisition of beta-lactamases (very rare) or PBP4/5 mutations [104]. Higher level of resistance in *E. faecium* has been attributed to over production of low affinity PBP-5, a protein that can take over the function of all PBPs [104]. A variety of point mutations have been described in both *E. faecium* and *E. faecalis* [104]. In addition, enterococci are “tolerant” to the activity of beta-lactams, and may appear susceptible in vitro but develop tolerance after exposure to penicillin. This property is an acquired characteristic. Enterococci quickly develop tolerance after exposure to as few as five doses of penicillin [105, 106].

Enterococci exhibit intrinsic low-level resistance to all aminoglycosides, precluding their use as single agents. Intrinsic resistance is attributed to an inability of the aminoglycoside to enter the cell (where they act by inhibiting ribosomal protein synthesis) [104]. While intrinsic mechanisms result in low-level aminoglycoside resistance, acquisition of mobile genetic elements typically underlies high-level aminoglycoside resistance in both *E. faecium* and *E. faecalis*. High-level resistance most frequently occurs through acquisition of a bifunctional gene encoding aph(2′′)-Ia-aac(6′)-Ie, which inactivates aminoglycosides [106]. However, several other genes have been identified that confer gentamicin resistance, including aph(2′′)-Ic, aph(2′′)-Id and aph(2′′)-Ib [107]. These genes are minor contributors to resistance compared to aph(2′′)-Ia-aac(6′)-Ie. Their prevalence varies by geographical region [104].

The acquisition of glycopeptides resistance by enterococci has seriously affected the treatment and control of these organisms [108]. Glycopeptides act by binding to the pentapeptide precursors of enterococci, thereby inhibiting cell wall synthesis. Glycopeptide-resistant organisms modify these pentapeptide precursors, which bind glycopeptides with 1000-fold lower affinity than normal precursors [104]. Various phenotypes of vancomycin-resistant enterococci (VRE) have been characterized; VanA and VanB operons are by far the most prevalent in human glycopeptide-resistant enterococci (GRE) infections [104]. GRE have emerged as a major cause of nosocomial infections. The majority of GRE infections have been attributed to *E. faecium*, though glycopeptide resistance occurs in *E. faecalis* and other *Enterococcus* species as well [109].

#### Antibiotic resistance in *Bacteroides fragilis*

*B. fragilis* is the most frequently isolated anaerobic bacteria, perhaps because it is both one of the most prominent in the intestinal microbiota, and is one of the easiest to culture in routine laboratory conditions. Beta-lactam antibiotics and 5-nitroimidazoles have been extensively used against anaerobic bacteria. The classical mechanisms of resistance to beta-lactams include production of beta-lactamases, alteration of penicillin-binding proteins (PBPs), and changes in outer membrane permeability to beta-lactams [110]. The most common mechanism at this time is inactivation by one of the various groups of beta-lactamases encoded by the *cepA* gene. Many beta-lactamases from the *B. fragilis* group are cephalosporinases that may be inhibited by lactamase inhibitors (clavulanic acid, and tazobactam). This explains the susceptibilities of many *Bacteroides* strains to the beta-lactam/beta-lactamase inhibitor combinations [111, 112].

Bacterial resistance to carbapenems arises because of the production of metallo-beta-lactamase encoded by the *cfiA* gene [113]. *cfiA* is normally poorly expressed. However, increased expression of *cfiA*, caused by the acquisition of an insertion sequence (IS) upstream of the gene, can lead to high-level carbapenem resistance.

Metronidazole, the first 5-nitroimidazole to be used clinically, was introduced in 1960, but it was not until 1978 that Ingham et al. reported the first clinical isolate of *B. fragilis* that was metronidazole-resistant after long-term therapy [114]. A wide range of metronidazole resistance mechanisms have been described in *B. fragilis* including decreased activity or total inactivation of electron transport chain components [115], overexpression of multidrug efflux pumps [116] and overexpression of DNA repair protein (RecA protein). However, the most common mechanism of resistance consists in the expression of 5-nitroimidazole nitroreductases (encoded by the *nimA*-*G* genes) which are located on the chromosome or on a plasmid and transform 4- or 5-nitroimidazole genes to 4- or 5-aminoimidazoles [115]. Metronidazole-resistant strains of the *B. fragilis* group have been described in several countries, but in general, resistance is low [117]. As routine susceptibility testing of anaerobic bacteria in most laboratories is only performed in blood or other severe infections, it is difficult to estimate how frequent MDR *B. fragilis* group strain is.

### Antibiotic resistance in intra-abdominal infections

*Surveillance studies can help clinicians to identify trends in pathogens incidence and antimicrobial resistance, including identification of emerging pathogens at national and global levels.*

*Some epidemiological studies have monitored antimicrobial resistance in IAIs identifying changes in resistance patterns, mostly of Gram negative bacteria.*

#### ESBL-producing *Enterobacteriaceae*

In the setting of intra-abdominal infections the main issue of resistance is due to ESBL producing *Enterobacteriaceae* [118]. Since 2002, the Study for Monitoring Antimicrobial Resistance Trends (SMART) has monitored the in vitro antibiotic susceptibility patterns of clinical Gram-negative collected worldwide from intra-abdominal cultures [119]. Limitations include the small number of contributing centers per country, and the characteristics of participating centers which are usually major teaching or tertiary-care centers.

From 2002 to 2011, the prevalence of MDR Gram negative bacilli, especially ESBL producers, has increased worldwide with regional variations in their distribution [119]. The prevalence of ESBLs producers in IAI isolates has steadily increased over time in Asia, Europe, Latin America, the Middle East, North America and the South Pacific. In contrast, the trend for ESBLs in intra-abdominal infection isolates from Africa has surprisingly, statistically significantly, decreased over time [119]. However only 7 African sites (3.9 %) (1 from Morocco, 2 from Tunisia and 4 from South Africa) were involved in the SMART Study.

Although ESBLs producing *Enterobacteriaceae* are common in hospital acquired IAIs, they are now being seen in community acquired IAIs as well as. In 2010, Hawser et al. [120] reported the incidence of ESBLs producers in community and hospital acquired IAI in Europe from 2002 to 2008. In order to differentiate community-acquired from hospital acquired IAIs, isolates were divided into those obtained from cultures collected <48 and ≥48 h after hospital admission, though this simple cut-off may result in mis-classification of some IAIs.

The SMART Study data showed a significant increase in ESBL-positive *E. coli* isolates (from 4.3 % in 2002 to 11.8 % in 2008 [*P* < 0.001]) with a smaller (and not statistically significant) increase in ESBL-positive *K. pneumoniae* isolates (increasing from 16.4 to 17.9 % [*p* > 0.05]) in Europe from 2002 to 2008. Hospital-acquired isolates were more common than community-acquired isolates, at 14.0 versus 6.5 %, respectively, for *E. coli* (*P* < 0.001) and 20.9 versus 5.3 %, respectively, for *K. pneumoniae* (*P* < 0.01) [120].

In the CIAOW Study [121] (Complicated intra-abdominal infections worldwide observational study), 68 medical institutions collaborated in a worldwide multi-center observational study, during a six-month study period (October 2012-March 2013). Among intra-operative isolates, ESBL producing *E. coli* isolates comprised 13.7 % (75/548) of all *E. coli* isolates, while ESBL producing *K. pneumoniae* isolates represented 18.6 % (26/140) of all *K. pneumoniae* isolates. ESBL producing *Enterobacteriaceae* were more prevalent in patients with healthcare-associated IAIs than they were in patients with community-acquired IAIs. Among healthcare associated infections, ESBL-positive *E. coli* isolates comprised 20.6 % (19/92) of all identified *E. coli* isolates, while ESBL-positive *K., pneumoniae* isolates made up 42.8 % (15/35) of all identified *K. pneumoniae* isolates.

#### *Klebsiella pneumoniae* Carbapenemases

*K. pneumoniae* carbapenemases (KPCs) are becoming an increasingly significant problem worldwide [122]. *E. coli* isolates from IAIs demonstrate consistently low resistance to carbapenems since the beginning of SMART. *K. pneumoniae* also continue to remain susceptible to carbapenems. Although carbapenem activity against *K. pneumoniae* from IAIs is also high, it is slightly lower than activity against *E. coli*. A total of 2841 clinical isolates of *K. pneumoniae* from intra-abdominal infections worldwide were collected in the SMART study during 2008 and 2009 [122]. Globally, 6.5 % of isolates were ertapenem resistant based on the June 2010 clinical breakpoints published by the Clinical and Laboratory Standards Institute, with MICs of ≥1 μg/ml [122].

#### *Pseudomonas aeruginosa*

*P. aeruginosa* was the third most common pathogen in IAIs at a rate of 5 % in the SMART Study [110]. In 2013, Babinchak et al. reported the trends in susceptibility of selected Gram-negative bacilli isolated from IAIs in North America from 2005 to 2010 [114]. The resistance of *P. aeruginosa* to fluoroquinolones significantly increased over time, from approximately 22 % in 2005, to 33 % in 2010. Imipenem activity remained unchanged with 20 % resistance. During this period, resistance to piperacillin-tazobactam, cefepime and ceftazidime remained unchanged at 23 to 26 %. [123]. However, the SMART Study demonstrated that the activity of select antimicrobials varied in different regions of the world. In South Africa during 2004–2009, *P. aeruginosa* resistance to piperacillin-tazobactam was 8 %, while it was approximately 25 % to cefepime, ceftazidime and imipenem, and 27 % to amikacin [119]. In China, during relatively the same time period (2002–2009), the resistance of *P. aeruginosa* to amikacin was 12 %, and to piperacillin-tazobactam was 8 % [119].

In the CIAOW study, among the microorganisms isolated from intraoperative samples, isolates of *P. aeruginosa* comprised 5.6 % of all aerobic identified bacteria. However, no significant differences between community acquired infections and healthcare associated infections (5.4 % in community acquired infections, versus 5.7 % healthcare associated infections) was demonstrated [121].

#### Enterococci

Among Gram-positive bacteria, enterococci play a significant role in IAI. Some studies have demonstrated poor outcomes among patients with documented enterococcal infections, particularly in those with post-operative IAI [124–127] where enterococci coverage should be always considered.

In 2012 the Dutch Peritonitis Study Group analyzing all patients from the RELAP trial found that the presence of only gram positive cocci, predominantly *Enterococcus* spp., was associated with worse outcome, although in secondary peritonitis microbial profiles did not predict ongoing abdominal infection after initial emergency laparotomy [128].

The Montravers EBIIA (Etude épidémiologique Bactério-clinique des Infections Intra-Abdominales) study [129] described the clinical, microbiological and resistance profiles of community-acquired and nosocomial IAI. This study reported an increase in the prevalence of nosocomial *E. faecalis* infections in patients (33 % in hospital-acquired infections, versus 19 % in community-acquired infections; *P* < 0.05). Although enterococci are found in community-acquired infections (11.8 %), they were far more prevalent in hospital-acquired infections (24.2 %).

In the CIAOW Study [121], among all the aerobic Gram-positive bacteria identified in the intraoperative samples, Enterococci (*E. faecalis and E. faecium*) were the most prevalent bacteria, representing 15.9 % of all aerobic isolates. Although Enterococci were also present in community-acquired infections, they were more prevalent in healthcare-associated infections (22.3 % in health-care IAIs versus 13.9 % in community-acquired infections).

#### Methicillin resistant *Staphylococcus aureus* (MRSA)

Methicillin resistant *Staphylococcus aureus* (MRSA) is not commonly isolated from patients with community-acquired intra-abdominal infection [121]. Although community-acquired MRSA has been reported in many settings, MRSA has less impact in community-acquired IAI. However, it should be always considered in the case of wound infections. MRSA should be suspected in patients with health care–associated IAI known colonized with the organism or who are at risk because of prior treatment failure and significant antibiotic exposure. The susceptibility pattern differs between community-acquired and hospital acquired MRSA.

#### *Salmonella typhi*

*S. typhi* infection may lead to diffuse peritonitis followed by ileal perforations in endemic countries [130].

The emergence of multidrug-resistant (MDR) typhoid fever is a major global health threat affecting many countries where the disease is endemic, such as countries in South-Central and Southeast Asia and many parts of Africa and Latin America [131]. In the past, *S. typhi* infections were routinely treated with chloramphenicol, ampicillin, or trimethoprim-sulfamethoxazole, but MDR to these antibiotics started to emerge in 1990 [132]. In response, a shift towards the prescription of fluoroquinolones or third-generation cephalosporins has occurred.

Singhal et al. [133] reported the trends in antimicrobial susceptibility of *S. typhi* from North India over a period of twelve years (2001–2012). In 852 isolates of *S. typhi*, a statistically significant decreased (*p* <0.001) resistance to chloramphenicol, ampicillin and and co-trimoxazole was observed. Resistance to nalidixic acid was found to be highest amongst all the antibiotics; it has been rising since 2005 and is presently 100 %. Ciprofloxacin resistance was relatively stable over the time period studied with a drastic increase from 5.8 % in 2008 to 10 % in 2009, since then it has increased in 2011–12 to 18.2 % [133].

#### *Bacteroides fragilis*

Anaerobes are the predominant components of the bacterial flora of normal human mucous membranes. *B. fragilis* strains are one of commonly isolated commensals in the setting of IAIs. Most clinical laboratories do not routinely perform the susceptibility testing of anaerobic isolates. In fact, their isolation requires appropriate methods of collection, transportation, and cultivation of specimens. Consequently, the treatment of anaerobic infections is often selected empirically [134].

*B. fragilis* strains are mostly sensitive to metronidazole, beta-lactam/beta-lactamase inhibitors and carbapenems. However, in the past years antibiotic resistance has increased among anaerobes and the susceptibility of anaerobic bacteria to antimicrobial agents has become less predictable [134].

Data of a national survey on antimicrobial resistance in *Bacteroides* strains*,* including 6574 isolates collected in 13 medical centers in United States from 1997 to 2007 were published in 2010 [135]. The study analyzed in vitro antimicrobial resistance to both frequently used and newly developed anti-anaerobic agents. Percent resistance was calculated using breakpoints recommended for the respective antibiotic by the CLSI or United States Food and Drug Administration. These data indicated that the carbapenems (imipenem, meropenem, ertapenem, and doripenem) and piperacillin-tazobactam were the most active agents against these pathogens, with resistance rates of 0.9–2.3 %. Metronidazole and tigecycline were the most active antibiotics among the non-beta-lactam agents. Metronidazole-resistant *Bacteroides* strains were also first reported during that period.

The susceptibilities of 824 *Bacteroides fragilis* group isolates against nine antibiotics were evaluated in a Europe-wide study involving 13 countries [136]. Piperacillin/Tazobactam was more active than amoxicillin/clavulanic acid (3.1 and 10.4 % resistance, respectively). Dramatic increases in resistance were observed for cefoxitin, clindamycin and moxifloxacin, with rates of 17.2, 32.4 and 13.6 %, respectively. The lowest resistances were found for imipenem, metronidazole and tigecycline (1.2, <1 and 1.7 %) [136].

### Antimicrobial stewardship

*Although most antimicrobial use occurs in the community, the intensity of use in hospitals is far higher; hospitals are therefore particularly important in the containment of antimicrobial resistance.*

*Hospital based Antibiotic Stewardship Programs (ASPs) can help clinicians both to optimize the treatment of infections and reduce adverse events associated with antibiotic use.*

*Given the urgent need to improve antimicrobial use in healthcare all acute care hospitals should implement Antibiotic Stewardship Programs.*

Antimicrobial stewardship is an emerging strategy designed to optimize outcomes and reduce the emergence of resistant organisms through the pillars of surveillance, infection control and optimizing the use of antimicrobial therapy. Educating clinicians in the appropriate use of antimicrobials is an essential facet of antimicrobial stewardship [117].

Core principles of antimicrobial stewardship include the use of antibiotic prophylaxis only when there is proven efficacy, use of the narrowest spectrum of antimicrobial therapy with proven efficacy, use of the least number of agents and for the shortest length of therapy to achieve efficacy, and appropriate antimicrobial dosing to maximize efficacy and limit complications. However, the best strategies for an antimicrobial stewardship program (ASP) are not definitively established and are likely to vary based on local culture, policy and routine clinical practice [15]. Observational data support a significant association between stewardship practices and reduction of antibiotic resistance. In a retrospective before and after study design, analysis of two ICUs within a single institution (trauma and surgical) before and after the implementation of service specific antibiotic stewardship protocols, Dortch et al. demonstrated a significant reduction in the percentage of multidrug resistant gram negative pathogens isolated and a corresponding decrease in broad spectrum antibiotic use [137].

The Infectious Diseases Society of America/Society for Healthcare Epidemiology of America (IDSA/SHEA) guidelines identify two core proactive evidence-based strategies and several supplemental strategies for promoting antimicrobial stewardship [1]: first, a proactive strategy of either formulary restriction or a requirement for pre-approval for specific drugs or both, and second, a strategy of performing prospective audit with intervention and feedback to the prescriber. Restriction of antimicrobial use may be obtained either by limited access to available antimicrobials in the hospital through restriction of the hospital formulary, or implementation of a requirement for preapproval and a justification for prescribing drugs on the restricted list. Both methods have been shown to be effective in reducing the use and costs of restricted antimicrobials [138].

To estimate the effectiveness of antimicrobial stewardship programs and evaluate their impact on the incidence of antimicrobial-resistant pathogens or *C. difficile* infection and on clinical outcome a Cochrane meta-analysis was performed in 2013 [139]. Eighty-nine studies were included. The meta-analysis showed that interventions to decrease excessive antibiotic prescribing for hospital inpatients reduced antimicrobial resistance and hospital-acquired infections. Interventions to increase effective prescribing improved clinical outcomes. These data supported the use of restrictive interventions in urgent cases. However, persuasive and restrictive interventions were equally effective after six months.

Restrictive interventions do seem to have a greater immediate impact than persuasive interventions. However, with the passage of time, prescribers often find ways to circumvent restrictions [139].

Prescribing is a complex social process. Restriction is useful in urgent situations, but because of the reduced effects over time, programs and strategies should be balanced with positive actions. The ultimate goal of a stewardship should be to stimulate a behavioral change in prescribing practice.

In this context, education of prescribers is crucial to convince clinicians to use antibiotics judiciously.

The supplemental strategies employed in ASP include, implementation of guidelines and clinical pathways, antimicrobial order forms, streamlining or de-escalation, combination therapy, dose optimization, and IV-to-PO switch, therapeutic substitution, cycling, mixing and use of computer decision support. In general, several of these strategies are implemented in the daily practice simultaneously with one or both of the two core strategies.

### Management of intra-abdominal infections

*The treatment of patients with complicated IAI involves both timely source control and antimicrobial therapy.*

*Empiric antimicrobial therapy is important in the management of intra-abdominal infections and must be broad enough to cover all likely organisms. Adequate source control is mandatory in the management of complicated IAIs.*

The treatment of patients with complicated IAI involves both source control and antimicrobial therapy. Source control encompasses all measures undertaken to eliminate the source of infection, reduce the bacterial inoculum and correct or control anatomic derangements to restore normal physiologic function [140]. Inadequate source control has been associated with increased mortality in patients with complicated IAI [141, 142]. Surgical source control entails resection or suture of a diseased or perforated viscus (e.g. diverticular perforation, gastroduodenal perforation), removal of the infected organ (e.g. appendix, gall bladder), debridement of necrotic tissue, resection of ischemic bowel and repair/resection of traumatic perforations, or drainage of infected fluid collections. The source control procedure will depend on the patient characteristics, organ affected, and specifically on the pathology that is encountered.

Ultrasound and CT guided percutaneous drainage of abdominal abscesses is safe and effective in selected patients [143–146], with 62–91 % cure rates and with morbidity and mortality rates equivalent to those of surgical drainage. Recent advances in interventional and more aggressive source control techniques, such as open abdomen strategy [147], could improve the outcome of patients with severe complicated IAI [148].

Although new surgical techniques, supported by innovative technology, have improved treatment for these patients, the markedly reduced development of new antibiotics has been unable to match the rapidly increasing phenomena of antimicrobial resistance making it a major ongoing challenge associated with the management of complicated IAI. Antimicrobial therapy plays an integral role in the management of complicated IAI. The main objectives of antimicrobial therapy in the treatment of IAI are to prevent local and haematogenous spread, and to reduce late complications.

### Classifications

*The term intra-abdominal infections (IAI), describes a wide heterogeneity of patient populations. A complete classification that includes all aspects of intra-abdominal infections does not exist. An ideal classification guiding clinicians in treatment should include:*

- *the origin of source of infection;*
- *the anatomical extent of infection;*
- *the presumed pathogens involved and risk factors for major resistance patterns; and*
- *the patient's clinical condition.*

IAI encompass a variety of pathological conditions, ranging from uncomplicated appendicitis to faecal peritonitis. IAI are usually classified either as uncomplicated or complicated [149].

In uncomplicated IAI, the infection only involves a single organ and does not extend to the peritoneum. Such patients can be managed by either surgical resection or antibiotics [150]. In complicated IAI, the infectious process extends beyond the organ, causing either localized or diffuse peritonitis [150]. These situations require both source control and antimicrobial therapy.

A universally accepted classification divides infective peritonitis into primary peritonitis, secondary peritonitis and tertiary peritonitis [151]. Primary peritonitis is a diffuse bacterial infection (usually single organism) without loss of integrity of the gastrointestinal tract, typically seen in cirrhotic patients with ascites or patients with an indwelling peritoneal dialysis catheter. Secondary peritonitis, the most common form of peritonitis (>90 % cases), is an acute peritoneal infection resulting from loss of integrity of the gastrointestinal tract [152]. Examples include visceral perforations or necrosis of the gastrointestinal tract, blunt or penetrating trauma, and post-operative leakage of anastomoses or suture lines. Tertiary peritonitis is defined as a recurrent infection of the peritoneal cavity that occurs >48 h after apparently successful and adequate surgical source control of secondary peritonitis [153–155]. It is more common among critically ill or immunocompromised patients and may often be associated with highly resistant pathogens including *candida spp*. It is typically associated with high morbidity and mortality. Although tertiary peritonitis has been accepted as a distinct entity [153–155], it represents an evolution and complication of secondary peritonitis, therefore the term “ongoing peritonitis” may better indicate that it is not a different disease than secondary peritonitis, but rather represents secondary peritonitis lasting longer and harbouring other (selected) pathogen.

Traditionally, infections have been classified as, either community-acquired or hospital-acquired, dependent on the place of acquisition [155]. Hospital-acquired intra-abdominal infections (HA-IAI) are often associated with surgery or another invasive procedure (gastrointestinal endoscopy, invasive radiology). The most frequent type of HA-IAI is post-operative peritonitis (PP) [156–160], is the most common cause of which is anastomotic leakage [158]. In rare conditions, HA-IAI can occur in patients hospitalized for reasons unrelated to abdominal pathology and no prior abdominal surgery [161].

The term “healthcare-associated infection” (HCAI) is a new term for infections acquired during the course of receiving healthcare [162]. It includes hospital-acquired infections but also infections in patients living in skilled nursing facilities, having recent hospitalization within 90 days, using aggressive medical therapies (intravenous therapy, wound dressing) at home and invasive therapies (haemodialysis, chemotherapy, radiotherapy) in outpatient clinics within 30 days of the index infection [162].

However, in the years after the first proposal by Friedman et al. [162], a consensus definition of HCAI has not been reached. A systematic review of all definitions of HCAI used in clinical studies was published in 2014 [163]. The initial definition of HCAI seems to be increasingly accepted: “Attendance at a hospital or haemodialysis clinic in the previous 30 days and residence in a nursing home or long-term care facility” [163].

Differentiating community-acquired intra-abdominal infection (CA-IAIs) and healthcare-associated intra-abdominal infections (HCA-IAIs) is useful to define the presumed resistance patterns and specify patients with increased likelihood of infection caused by MDRO. Among patients with HCAI, those with hospital-acquired infections may be associated with increased mortality due to underlying patient health status and severity criteria at the time of diagnosis.

Grading of the clinical severity of patients with complicated IAI has been be well described by the sepsis definitions. Sepsis is a complex, multifactorial syndrome that may develop into conditions of varying and escalating severity [163–165].

Mortality rates increase in patients developing organ dysfunction and septic shock [166]. Mortality of septic patients from abdominal origin has decreased due to advances in management of the underlying infection and support of failing organs, but is still high [167]. The CIAOW study [121] described the epidemiological, clinical, and treatment profiles of complicated IAI worldwide. The overall mortality rate was 10.5 %, while it was significantly higher (36.5 %) for patients with organ dysfunction or septic shock at admission.

Recently, sepsis definitions were revised. Sepsis is defined as life-threatening organ dysfunction caused by a dysregulated host response to infection. Septic shock is defined as a subset of sepsis in which circulatory, cellular, and metabolic abnormalities are associated with a greater risk of mortality than with sepsis alone and should be clinically identified by a vasopressor requirement to maintain a mean arterial pressure of 65 mmHg or greater, and serum lactate level greater than 2 mmol/L (>18 mg/dL) in the absence of hypovolemia. Under this redefined terminology, “severe sepsis” has been abandoned [168].

### Antimicrobial selection

*Initial antimicrobial therapy for patients with IAIs is empiric in nature because critically-ill patients need immediate treatment and microbiological data (culture and susceptibility results) usually requires ≥24 h for the identification of pathogens and antibiotic susceptibility pattern.*

Antimicrobials should be used after a treatable IAI has been recognized or when there is a high degree of suspicion for infection. Initial antimicrobial therapy in patients with IAIs is typically empirical in nature because they need immediate treatment (especially in critically-ill patients), and microbiological data (culture and susceptibility results) usually requires ≥24 h for the identification of pathogens and antibiotic susceptibility patterns. However, in non-critically ill patients survival benefit from adequate empiric antimicrobial therapy has not been consistently demonstrated, even in patients with Gram-negative bacteremia [169].

Knowledge of local rates of resistance and the risk factors that suggest an MDRO should be involved as essential components of the clinical decision-making process when deciding on which antimicrobial regimen to use for empiric treatment of infection [1]. Every clinician starting empiric therapy should know the local epidemiology. Surveillance initiatives are important, both in a local and in a global context. If local epidemiology suggests that a patient has been infected with a strain already known to be resistant to antibiotics, then inappropriate antimicrobial therapy, which fails to cover known resistance patterns risks further disruption of the natural flora and selecting for resistant variants without providing effective treatment.

Hospitals in the United States are required to publish an annual antibiogram that may be used as a source guideline for selecting appropriate antibiotics based on local resistance/susceptibility data.

### Published guidelines

*Different sets of guidelines for the management of patients with IAIs have been published.*

*Guidelines have a great impact on clinical care. They should incorporate stewardship principles.*

Different sets of guidelines outlining the clinical management of IAIs have been published [170–178].

Historically, treatment guidelines have not taken into consideration antimicrobial stewardship principles when setting the priority order of antimicrobial options, and instead have focused primarily on safety and efficacy data.

Guidelines have major impact on delivery of clinical care, and on regulatory review of hospital performance. Hopefully, these guidelines will evolve to incorporate stewardship principles, in addition to safety and efficacy, when setting the priority order for recommended antimicrobial therapies.

### Antimicrobial selection

#### Antimicrobial selection in community-acquired infections

*For patients with CA-IAI, antimicrobial agents with a narrow spectrum of activity encompassing all likely organisms should be administered. However, a patient with risk factors for ESBL infection who is hemodynamically unstable may warrant empiric therapy to cover for ESBLs, with plans to de-escalate therapy once microbiology is known.*

The major pathogens involved in community-acquired IAI (CA-IAI) are likely to be due to a patient’s own flora. Therefore, they are predictable and include *Enterobacteriaceae* (predominantly *E. coli* and *Klebsiella* species), viridans group streptococci, and anaerobes (especially *B. fragilis*). For patients with CA-IAI, antimicrobial agents with a relatively narrower spectrum of activity encompassing wild-type strains from the above-mentioned species should be administered. However, if patients with CA-IAI have risk factors for infections due to ESBL producing *Enterobacteriaceae*, and in particular if the patient is hemodynamically unstable, antimicrobial agents that are effective against ESBLs may be warranted. Clinical stability and physiological well-being is an important factor, as compromised patients will suffer increased morbidity and mortality if initial therapy is ineffective [179]. In contrast, less severely ill patients may have more time for the clinician to know that initial therapy was not active. Specific risk factors for ESBL phenotype among infecting pathogens includes recent exposure to antibiotics (particularly beta-lactams or fluoroquinolones) within 90 days of IAI or known colonization with ESBL producing *Enterobacteriaceae,* [180, 181]. An additional risk may be represented by a recent trip to a region where MDR *Enterobacteriaceae* are widespread in the community [182].

#### Antimicrobial selection in health-care associated infections

*For patients with HCA-IAI, empiric antimicrobial regimens with broader spectra of activity should be administered as these patients have a higher risk of infections due to MDRO.*

By contrast, for patients with HCA-IAI, antimicrobial regimens with broader spectra of activity are preferable, as those patients have a higher risk of infections due to MDROs [183]. On receiving results of susceptibility testing, the clinician should opt for a narrow spectrum antimicrobial agent, which covers the likely causative organism. De-escalation of therapy must be weighed against the clinical significance of the culture results received as well as local epidemiology [184].

#### Antibiotic selection in critically Ill patients

*An inadequate empiric antimicrobial regimen is associated with unfavorable outcomes in critical ill patients. In these patients the following strategies should be always implemented to obtain an optimal response to therapy:*

- *early source control procedures when indicated;*
- *early initiation of therapy (ideally, within 1 h);*
- *correct dosing;*
- *considering risk factor for MDRO; and*
- *avoiding use of identical antibiotic and the same antibiotic class administered in the preceding 3 months.*

Infections are among the main factors contributing to mortality in intensive care units (ICU) [185]. Abdominal sepsis is a common indication for admission to the ICU. The abdomen is the second most common site of invasive infection among critically ill patients [186]. In 2014, the EPIC II study [187], including 13,796 adult patients from 1265 ICUs in 75 countries, revealed that mortality rate in patients who developed abdominal infections was significantly higher than in patients who had other infections (most of which were respiratory infections). Disease severity, need for organ support, and presence of co-morbidities were independently associated with mortality. In patients with organ dysfunction from septic shock, early appropriate empiric antimicrobial therapy has a significant impact on the outcome, independent of the site of infection [188]. The Surviving Sepsis Campaign guidelines recommend intravenous antimicrobials within the first hour of onset of sepsis and septic shock and the use of broad-spectrum agents with adequate penetration of the presumed site of infection [189]. An ineffective or otherwise inadequate antimicrobial regimen is one of the variables more strongly associated with unfavorable outcomes in critical ill patients [190]. Empiric antimicrobial therapy should be started as soon as possible in patients with organ dysfunction and septic shock [191–193].

#### De-escalation

The evidence on de-escalation strategies is largely restricted to patients with ventilator-associated pneumonia (VAP) [194–196] and patients with severe sepsis and septic shock, particularily in patients with IAIs [197, 198]. In 2013, a Cochrane review on de-escalation of antimicrobial treatment for adults with sepsis did not find adequate evidence to support this [199]. In 2014, a multicenter randomized trial investigating a strategy based on de-escalation of antibiotics resulted in prolonged duration of ICU stay and did not affect the mortality rate [198]. Several authors have associated antimicrobial de-escalation interventions in critically ill patients with reductions in length of hospitalization, inpatient antimicrobial use, adverse events, cost, and recovery of antimicrobial-resistant microorganisms [200, 201]. The safety and beneficial outcomes of carbapenem de-escalation as part of an antimicrobial stewardship program in an ESBL-endemic setting, was also recently confirmed [202].

### Antibiotic armamentarium

*The choice of empiric antibiotics in patients with IAI should be based on the severity of the infection, the individual risk for infection by resistant pathogens, and the local resistance epidemiology. Amoxicillin/clavulanate or cephalosporins in combination with metronidazole, are still good options for the treatment of non-severe IAIs, with piperacillin/tazobactam being a better choice if P. aeruginosa coverage is needed. The use of carbapenems should be limited so as to preserve activity of this class of antibiotics because of the concern of emerging carbapenem-resistance. Ciprofloxacin and levofloxacin are no longer appropriate first-line choices for empiric treatment in many regions because of the prevalence of fluoroquinolone resistance. Other options include aminoglycosides, particularly for suspected infections by Gram negative bacteria, and tigecycline especially when MDRO are suspected, though caution is advised for the latter, in the situation of a bacteremia.*

*Recent challenges in the management of multi-drug resistant Gram-negative infections, especially in critically ill patients, have reviewed the use of “old” antibiotics, such as polymyxins and fosfomycin.*

*Ceftolozone/tazobactam and ceftazidime/avibactam are new antibiotics that have been approved for treatment of cIAI infections (in combination with metronidazole) including infection by ESBLs and P. aeruginosa, though their role for the empirical therapy remains to be defined.*

IAI may be managed by either single or multiple antimicrobial regimens. Table 1 presents the spectrum of activity of antimicrobial agents for common IAI pathogens, Table 2 presents recommended intravenous antimicrobial doses for patients with IAI and preserved renal function. These doses may not be adequate for patients with morbid obesity as there are currently no specific dosing recommendations for antibiotics in obese patients [203].

**Table 1:** Antibiotics for treating patients with intra-abdominal infections based upon susceptibility. Use local antibiogram data for choosing optimal antibiotics in target population

Antibiotic	Enterococci	Ampicillin-resistant enterococci	Vancomycin-resistant enterococci	Enterobacteriaceae	ESBL-producing *Enterobactericeae*	*Pseudomonas aeruginosa*	Anaerobic Gram-negative bacilli
Penicillins/Beta-lactamase Inhibitors							
Amoxicillin/clavulanate	+	−	−	+	−	−	+
Ampicillin/Sulbactam	+	−	−	+	−	−	+/−
Piperacillin/tazobactam	+	−	−	+	+/−	+	+
Carbapenems							
Ertapenem	−	−	−	+	+	−	+
Imipenem/cilastatin	+/−^a^	−	−	+	+	+	+
Meropenem	−	−	−	+	+	+	+
Doripenem	−	−	−	+	+	+	+
Fluoroquinolones							
Ciprofloxacin	−	−	−	+	−	+^b^	−
Levofloxacin	+/−	−	−	+	−	+/−	−
Moxifloxacin	+/−	−	−	+	−	−	+/−
Cephalosporins							
Ceftriaxone	−	−	−	+	−	−	−
Ceftazidime	−	−	−	+	−	+	−
Cefepime	−	−	−	+	+/−	+	−
Ceftolozane/tazobactam	−	−	−	+	+	+	−
Ceftazidime/avibactam	−	−	−	+	+	+	−
Aminoglycosides							
Amikacin	^c^	^c^	^c^	+	+	+	
Gentamicin	^c^	^c^	^c^	+	+	+	−
Glycylcyclines							
Tigecycline	+	+	+	+^d^	+	−	+
5-nitroimidazole							
Metronidazole	−	−	−	−	−	−	+
Polymyxin							
Colistimethate (Colistin)	−	−	−	+^e^	+	+	−
Glycopeptides							
Teicoplanin	+	+	−	−	−	−	−
Vancomycin	+	+	−	−	−	−	−
Oxazolidines							
Linezolid	+	+	+	−	−	−	−

^a^“Imipenem/cilastatin” is more active against ampicillin-susceptible enterococci than ertapenem, meropenem and doripenem

^b^Ciprofloxacin is more active against *Pseudomonas aeruginosa* than levofloxacin

^c^Active in synergy with other agents

^d^Not active against *Proteus, Morganella* and *Providencia*

^e^Not active against *Morganella, Proteus, Providencia and Serratia*

**Table 2:** Recommended intravenous doses of the most commonly used antibiotics for patients with intra-abdominal infections and normal renal function (CrCl > 90 mL/min)

Intravenous Antibiotic	Intravenous dosing recommendation for patients with normal renal function*(CrCl > 90 mL/min)
Penicillins/ Beta-lactamase Inhibitors	
Amoxicillin/clavulanate	1.2 g 8-hourly
Ampicillin/Sulbactam	3 g 6-hourly
Piperacillin/tazobactam	4.5 g 6- 8-hourly or 3.375 g 6-hourly
Carbapenems	
Ertapenem	1 g 24-hourly
Imipenem/cilastatin	0.5 g 6-hourly (or1 g 8-hourly)
Meropenem	1 g 8-hourly
Fluoroquinolones	
Ciprofloxacin	400 mg 8–12 hourly
Levofloxacin	750 mg 24-hourly
Moxifloxacin	400 mg 24-hourly
Cephalosporins	
Ceftriaxone	1–2 g 24-hourly
Ceftazidime	2 g 8-hourly
Cefepime	1–2 g 8 hourly
Ceftolozane/tazobactam	1.5 g 8-hourly
Ceftazidime/avibactam	2.5 g 8-hourly
Glycylcyclines	
Tigecycline	100 mg initial dose, then 50 mg 12-hourly
Aminoglycosides	
Amikacin	15–20 mg/kg 24-hourly
Gentamicin	5–7 mg/kg 24-hourly
5-nitroimidazole	
Metronidazole	500 mg 6–8 hourly
Glycopeptides	
Teicoplanin	12 mg/kg 12-hourly times 3 loading dose then 12 mg/kg 24-hourly
Vancomycin	15–20 mg/kg/dose 8–12 hourly; in critically ill patients 25–30 mg/kg loading dose
Oxazolidinonees	
Linezolid	600 mg 12 hourly
Polymyxins	
Colistin	US: 2.5 to 5 mg/kg CBA 8–12 hourly Europe: 9 million IU 8–12 hourly as a slow intravenous; in critically ill patients 9 million IU loading dose as a slow intravenous infusion

Note–the above table provides general information, the susceptibility profile of individual organisms should be confirmed to guide antimicrobial therapy in all situations. Dosage should be adjusted according to the antibiotic's pharmacokinetic/pharmacodynamic profile in each patient

Higher dosages may be used in septic shock

#### Beta-lactam/beta-lactamase inhibitor combinations

Beta-lactam/beta-lactamase inhibitor combinations (BLBLI), including ampicillin/sulbactam, amoxicillin/clavulanate, ticarcillin/clavulanate, piperacillin/tazobactam, have an in vitro activity against Gram-positive, Gram-negative and anaerobe organisms [204]. However, increasing antimicrobial resistance to ampicillin/sulbactam and amoxicillin/clavulanate among *E. coli* and other *Enterobacteriaceae* including community-acquired isolates, during the last decade, has compromised clinical utility of these agents for empirical therapy of serious Gram-negative infections [205, 206]. This is likely due to excessive use of amoxicillin and amoxicillin-clavulanate in both children and adults, particularly in the treatment of upper respiratory tract infection. The combination of over use of these oral antibiotics in the community and potential for household transmission of resistant *E. coli* strains among family members make ampicillin/sulbactam and amoxicillin/clavulanate resistance unpredictable [207]. Fortunately, most isolates remain susceptible to other beta-lactam/beta-lactamase inhibitors such as piperacillin/tazobactam. Broad-spectrum activity of piperacillin/tazobactam, including anti-pseudomonal and anaerobic coverage, still make it an attractive option in the management of severe IAIs [208].

A meta-analysis of PubMed and Scopus databases providing data for mortality among patients treated with carbapenems, BLBLI or non- BLBLI (mainly cephalosporins and fluoroquinolones), preferably as monotherapy was published in 2013 [209]. The study reported no statistically significant difference in mortality between carbapenems and BLBLI administered as either empiric or definitive therapy. The authors concluded that the role of BLBLI should be further evaluated for definitive treatment [209]. In a recent study of 331 unique patients with ESBL bacteremia, piperacillin/tazobactam appeared inferior to carbapenems in the treatment of ESBL bacteremia [210]; the use of piperacillin/tazobactam in ESBLs infections is still controversial [211].

#### Cephalosporins

Most isolates of *E. coli* and other *Enterobacteriaceae* remain susceptible to third-generation cephalosporins. Among this drug class, cefotaxime, ceftriaxone and ceftizoxime, in combination with metronidazole may be options for empirical therapy of CA-IAI, due to the relatively narrow spectrum of coverage bacause these agents lack activity against *P. aeruginosa*. On the other hand, ceftazidime and cefoperazone have activity against *P. aeruginosa*, but have relatively less activity against streptococci as compared to other third-generation cephalosporins. Cefepime, a fourth-generation cephalosporin, with broader spectrum activity compared to ceftriaxone is a poor inducer of AmpC beta-lactamase, and is poorly hydrolyzed by the enzyme, allowing it to be effective against AmpC-producing organisms [66]. For empiric therapy, cefepime must be combined with metronidazole [212] because it does not possess anti-anaerobic activity.

The newest “5th” generation cephalosporins such as ceftobiprole have very broad-spectrum activity, exhibiting potent in vitro activity against a number of Gram-positive pathogens including MRSA, penicillin-resistant *Streptococcus pneumoniae*, and Gram-negative pathogens including AmpC producing *E. coli* and *P. aeruginosa*. Their role towards *E. faecium* PBP 5 (PBP 5fm) is controversial [213]. Ceftobiprole medocaril is approved for the treatment of community-acquired pneumonia and hospital-acquired pneumonia (excluding ventilator-associated pneumonia), in the European Union [214, 215]. It has not been approved for the treatment of cIAI.

#### Fluoroquinolones

Fluoroquinolones (FQ) have been widely used in the treatment of intra-abdominal infections because of their excellent activity against aerobic Gram-negative bacteria and tissue penetration [216]. Ciprofloxacin has in vitro activity against *P. aeruginosa*. Ciprofloxacin has lowest MIC against *P. aeruginosa* among commonly used fluoroquinolones such as levofloxacin and moxifloxacin.

Except for moxifloxacin, the FQ have a moderate activity against anaerobes and have been used in combination with metronidazole in the empiric treatment of IAI.

FQ are rapidly, and almost completely, absorbed from the gastrointestinal tract, particularly levofloxacin and moxifloxacin. Peak serum concentrations obtained after oral administration are very near those achieved with intravenous administration [217]. Prior patient use of FQ has been demonstrated as an independent risk factor for FQ resistance [218]. Therefore, the empiric use of FQ for IAI is discouraged in patients with recent exposure to this class of antibiotics. In addition, the increasing use of FQ in aged care facilities, particularly for the treatment of urinary tract infections, has contributed to the emergence of *E. coli* virulent strains, such as O25b-ST131, with substantially high FQ resistance rates in patients living at those facilities [219]. In recent years, resistance of *E.coli* to FQ has risen over time [218]. The worldwide increase in FQ resistance among *E. coli* and other *Enterobacteriaceasaee* has limited the non-stratified use of FQ for empirical treatment of IAI, particularly in critically-ill patients and those with HCA-IAI [206].

#### Carbapenems

For decades, carbapenems have been the antibiotics of first choice for ESBLs. The best option for targeting ESBLs (though with no coverage of *P. aeruginosa)* is ertapenem, a once daily administered carbapenem that otherwise shares the activity of imipenem, meropenem and doripenem against most species, including ESBL producing pathogens [220, 221]. Imipenem/cilastatin, meropenem and doripenem provide coverage for Gram-negative non-fermenting bacteria (e.g. *P. aeruginosa* and *A. baumannii*). However, inappropriate use of carbapenems should be avoided [222] because there is an association with the increase in carbapenem-resistant *Enterobacteriaceae*, e.g. the rapid spread of carbapenemases in *K. pneumoniae* or NDM-1 producing *Enterobacteriaceae* and *P. aeruginosa* [223].

Regarding *Enterococcus* coverage among carbapenems, impipenem/cilastatin is most active in vitro against ampicillin-susceptible *E. faecalis* while ertapenem, meropenem, and doripenem have limited activity against both *E. faecalis* and *E. faecium*. In addition, carbapenems are not generally recommended for use to treat bacteremia due to *Enterococcus* spp. In carbapenemase producing *K. pneumoniae* with an MIC ≤8 μg/ml*,* carbapenem-containing combinations, including meropenem or doripenem, is suggested [224].

#### Aminoglycosides

Aminoglycosides are particularly active against aerobic Gram-negative bacteria and act synergistically against certain Gram-positive organisms. They are effective against *P. aeruginosa,* but are ineffective against anaerobic bacteria. Because of their serious toxic side effects including nephrotoxicity and ototoxicity, some authors do not recommend aminoglycosides for the routine empiric treatment of community-acquired IAI. They may be reserved for patients with allergies to beta-lactam agents or when used in combination with beta-lactams for treatment of IAI secondary to MDRO [225]. However, other authors have questioned the clinical importance of the toxic side-effects [226], and their decreased activity in acidic environment such as pus. In any case, this class of antibiotics remains an important option in the antimicrobial armamentarium for combating Gram-negative bacteria and widening the spectrum of the empirical therapy when MDRO are suspected [178].

#### Tigecycline

Tigecycline, an antibiotic from the group of the tetracyclines, does not feature in vitro activity against *P. aeruginosa* or certain *Enterobacteriaceae* (*Proteus spp*., *Serratia spp*., *Morganella morganii*, *Providencia stuartii*). However, it remains a viable treatment option for complicated IAI due to its favorable in vitro activity against anaerobic organisms, enterococci, several ESBLs and some strains of carbapenemase-producing *Enterobacteriaceae* [227]. In several trials, excess mortality was seen in patients treated with tigecycline when compared with other drugs; in 12 of 13 phase 3 and 4 comparative clinical trials [228], all-cause mortality was found higher in the tigecycline group versus the comparison group. Study-level and patient-level analyses identified that patients in the hospital-acquired pneumonia trial, particularly those with ventilator-associated pneumonia with baseline bacteremia, were at a higher risk of clinical failure and mortality.

A mortality analysis was used to investigate the association of baseline factors in abdominal infections, including severity of illness at study entry and treatment assignment, with clinical failure and mortality. Mortality modelling identified multiple factors associated with death which did not include tigecycline and which were forced into the model.

Similarly, attributable mortality, among subjects who died of primary infection, in the cIAI studies, showed no difference among treatments. Combined with the four phase 3 and 4 cIAI trials that demonstrated the non-inferiority of tigecycline to the comparator regimens, these results suggest that deaths were less related to clinical failure and that other factors or patient co-morbidities were more likely to contribute to death [229].

Because of poor plasma concentration tigecycline performs poorly in bacteremic patients, with a much higher risk of failing clear bacteremia than the comparator. Tigecycline should not be considered first line for health care associated pneumonia, bacteremia or endocarditis. Nonetheless, tigecycline remains an important treatment option for patients with complicated IAI [230, 231]. Recently, tigecycline has been become an important option in managing infections due to MDR Gram negative bacteria [232] including NDMs, KPCs, and other carbapenemases, as some of these pathogens remain susceptible to tigecycline. However, high failure rates in cases of monotherapy with this antibiotic have occurred implying that combinations therapy should be recommended [233]. Higher-dose regimens have been associated with better outcomes than conventional administration due to Gram-negative MDR bacteria in a cohort of critically ill patients with severe infections [234].

#### Polymixins

Polymyxins, discovered in 1940s, are a group of polycationic peptide antibiotics that exhibit potent efficacy against most gram-negative bacteria. Among all the five chemical compounds (A–E) of polymyxins, only polymyxin B and E (colistin) are clinically used. Since the 1970s, these preparations were practically abandoned because of reports of severe adverse events. However, recent challenges in the management of multi-drug resistant (MDR) Gram-negative infections, especially in critically ill patients, have revived the clinical use of polymyxins [235–237]. The nephrotoxicity and neurotoxicity of polymyxins has been the major limiting factor in their clinical application. Challenging these earlier concerns, recent data, mainly from cases series, demonstrate that the use of polymyxins is relatively safe provided that recommended dosages are used and renal function is closely monitored [235, 238, 239].

Recently The US Food and Drug Administration (FDA) and European Medicines Agency (EMA) have approved updated dose recommendations for intravenous colistin in patients with various degrees of renal function [240].

The EMA recommendations were based on a review of the available clinical, pharmacological and pharmacokinetic data.

EMA recommended expression of colistin dose in IU of colistimethate sodium. Based on the limited available evidence the recommended dose in adults was 9 million IU (approximately 300 mg) daily in 2 or 3 divided doses as a slow intravenous infusion; in critically ill patients a loading dose of 9 million IU was suggested. EMA suggested to reduce dosage according to creatinine clearance in patients with renal impairment.

Also US Food and Drug Administration (FDA) approved changes to the dosage and administration section of the product label for colistimethate in the United States. The recommended dose was 2.5 to 5 mg/kg colistin base activity (CBA) per day in 2 to 4 divided doses for patients with normal renal function, depending on the severity of the infection. A loading dose was not recommended for critically ill patients. The FDA recommended dosing regimen accounts for renal function. The most significant difference regards loading dose suggested in the EU but not in the United States’ recommendations.

#### Fosfomycin

Renewed interest in ‘old’ antibiotics has also focused interest in Fosfomycin. While traditionally fosfomycin disodium was administered parenterally, several countries have recently approved the oral administration of fosfomycin tromethamine for treating urinary tract infections (UTIs) caused by *Escherichia coli* and *E. faecalis*. Its use as a single agent is usually restricted in critically ill patients. However, intravenous fosfomycin has been administered in combination with other antibiotics for the treatment of MDR Gram-positive and Gram-negative bacteria including KPC [241].

The daily dose of intravenous fosfomycin disodium ranges from 12 to 16 g on average, administered in 2–4 infusions. Renal impairment significantly decreases the excretion of fosfomycin. For intravenous administration of fosfomycin, the doses should be reduced if the creatinine clearance is less than 50 ml/min [242].

The primary limitations of fosfomycin are the lack of established regimens for complicated infections and the lack of availability of the intravenous formulation in many countries [243].

#### New antibiotics

Ceftolozane/tazobactam [244, 245] and ceftazidime/avibactam [246, 247] have recently been approved in some national agencies for the treatment of intra-abdominal infections. By adding beta-lactamase inhibitor (tazobactam or avibactam), these new agents have strong activity against MDR Gram-negative pathogens. Unlike other beta-lactam and beta-lactamase inhibitor combination agents, these new agents should be combined with metronidazole for complicated IAI due to limited activity against some *Bacteroides* species.

These antibiotics will be valuable for treating infections caused by MDR Gram-negative bacteria in order to preserve carbapenems. Notably, ceftazidime/avibactam has demonstrated consistent activity against KPC-producing organisms [248].

In some instances that resistance can emerge to new antibiotics very rapidly after their first clinical trials, treatment with these new agents should be done in parallel with continued susceptibility testing [249]. Although many reviews have been written, their precise role as an empiric treatment for complicated IAI remains to be defined [250]. Cautious clinical use is advised, until their precise roles are further defined as empirical treatment.

### The effect of fungal involvement in IAI

*Empiric antifungal therapy should be considered in patients with clinical evidence of intra-abdominal infection and significant risk factors for candidiasis:*

- *recent abdominal surgery;*
- *anastomotic leaks;*
- *necrotizing pancreatitis; and*
- *failure of treatment for bacterial infections.*

The epidemiological role of *Candida spp.* in IAI has not yet been conclusively defined [251]. However, recent data suggest that some specific subpopulations are at higher risk of fungal involvement, (i.e. complicated cases of bariatric surgery). In a recent study, Zappella et al. [252] reported 41 % of candida-positive patients with postoperative peritonitis following bariatric surgery. Isolation of *Candida* spp. in samples from IAI is associated with poor outcomes. In an observational study, Montravers et al. showed that isolation of *Candida* spp. was an independent risk factor of mortality in nosocomial peritonitis patients (odds ratio, 3; 95 % confidence interval, 1.3-6.7, *p* < 0.001). Antifungal treatment did not improve survival [253]. Recently, IDSA guidelines for the treatment of invasive candidiasis were developed and explicitly addressed candidal peritonitis [254]. Clinical evidence supporting the use of antifungal therapy for patients with suspected intra-abdominal invasive candidiasis is limited. Most studies are small and uncontrolled, single-center, or performed in specific patient cohorts. IDSA guidelines suggested considering empiric antifungal therapy for patients with clinical evidence of intra-abdominal infection and significant risk factors for candidiasis, including those with recent abdominal surgery, anastomotic leaks, or necrotizing pancreatitis, who are doing poorly despite treatment for bacterial infections.

Several meta-analyses of antifungal prophylaxis in high-risk surgical ICU patients have yielded conflicting results [255–258].

For the majority of ICU patients at high invasive candidiasis risk, a preemptive antifungal strategy, based on clinical risk factors and microbiologic evidence of substantial colonization, has been proposed [259].

A recent randomized, double-blind, placebo-controlled trial assessed a preemptive antifungal approach with an echinocandin in intensive care unit patients requiring surgery for intra-abdominal infection [260].

The study was unable to provide evidence that preemptive administration of an echinocandin was effective in preventing IC in high-risk surgical intensive care unit patients with intra-abdominal infections.

Preferred empiric therapy in critically ill patients or those previously exposed to an azole is an echinocandin (caspofungin: loading dose of 70 mg, then 50 mg daily; micafungin:100 mg daily; anidulafungin: loading dose of 200 mg, then 100 mg daily). However, fluconazole, 800-mg (12 mg/kg) loading dose, then 400 mg (6 mg/kg) daily, should be still considered first-line antifungal therapy, in hemodynamically stable patients who are colonized with azole susceptible Candida species or who have no prior exposure to azoles.

The duration of therapy should be determined by adequacy of source control and clinical response.

A role for echinocandin in the management of critically ill patients is confirmed by the increasing incidence of fluconazole-resistant and susceptible-dose dependent strains that should be taken into account when selecting empiric therapy in patients with severe sepsis or septic shock [261, 262].

### Dosage

*Knowledge of the pharmacokinetic and pharmacodynamic antimicrobial properties of each antimicrobial inform rational dosing.*

*Optimal use of the pharmacokinetic/pharmacodynamic characteristics of antimicrobial agents is important for obtaining good clinical outcomes and reduction of resistance.*

The antimicrobial dosing regimen should be established depending on host factors and properties of antimicrobial agents. Antimicrobial pharmacodynamics integrates the complex relationship between organism susceptibility and patient pharmacokinetics. Pharmacokinetics describes the fundamental processes of absorption, distribution, metabolism, and elimination and the resulting concentration-versus-time profile of an agent administered in vivo. The achievement of appropriate target site concentrations of antimicrobials is essential to eradicate the relevant pathogen. Suboptimal target site concentrations may have important clinical implications, and may explain therapeutic failures, in particular, for bacteria for which in vitro MICs are high [263]. Antimicrobials typically need to reach a site of action outside the plasma. This requires the drug to pass through the capillary membranes. Disease and drug-related factors can contribute to differential tissue distribution [264]. Concentration gradient between the plasma and the peritoneal space has been studied for some antibiotics and have shown large variability [265–268]. Commonly encountered situations where pharmacokinetics change and dosing individualization may be necessary include renal and hepatic dysfunction. Dose reductions may be necessary to prevent accumulation and toxicity in patients with reduced renal or hepatic function.

Knowledge of the pharmacokinetic and pharmacodynamic antimicrobial properties of each drug including (inhibition of growth, rate and extent of bactericidal action, and post-antibiotic effect) may provide a more rational determination of optimal dosing regimens in terms of the dose and the dosing interval [269]. Optimal use of the pharmacokinetic/pharmacodynamic relationship of anti-infective agents is important for obtaining good clinical outcomes and reduction of resistance [270]. Dosing frequency is related to the concept of time-dependent versus concentration-dependent killing. Beta-lactams exhibit time-dependent activity and exert optimal bactericidal activity when drug concentrations are maintained above the MIC [271, 272]. Therefore, it is important that the serum concentration exceeds the MIC for appropriate duration of the dosing interval for the antimicrobial and the organism. Higher frequency dosing, prolonged infusions and continuous infusions have been utilized to achieve this effect [267]. For beta lactams, prolonged or continuous infusions have been advocated in order to maximize the time that the drug concentration exceeds the MIC, whereas high peak concentrations are not beneficial. However, large randomized controlled trials comparing continuous with intermittent infusion of piperacillin/tazobactam in patients with complicated IAI [273] as well as piperacillin/tazobactam, ticarcillin/clavulanate or meropenem in patients with severe sepsis [274], did not demonstrate different outcomes. These results may not be generalizable to patients with high severity of illness and infections caused by less susceptible pathogens with high MIC (e.g. *P. aeruginosa*), for which the greatest potential for a clinically relevant benefit is predicted by pharmacokinetic/pharmacodynamic theory, and has been supported by some retrospective studies [275, 276]. Prolonged or continuous infusions of beta lactams should therefore be considered for the treatment of critically ill patients with hospital-acquired IAI.

In contrast, antibiotics such as aminoglycosides exhibit concentration-dependent activity and should be administered in a once daily manner (or with the least possible number of daily administrations) in order to achieve high peak plasma concentrations.

With these agents, the peak serum concentration, and not the time the concentration remains above the MIC, is more closely associated with efficacy [271, 272]. In terms of toxicity, aminoglycosides nephrotoxicity is caused by a direct effect on the renal cortex and its uptake saturation. Thus, an extended interval dosing strategy reduces the renal cortex exposure to aminoglycosides and reduces the risk of nephrotoxicity [277].

In patients with septic shock, administering an optimal first dose is probably as equally important as to the timing of administration [271]. This optimal first dose could be described as a loading, or front-loaded dose and is calculated from the volume of distribution (Vd) of the drug and the desired plasma concentration. The Vd of hydrophilic agents (which disperse mainly in water such as beta-lactams, aminoglycosides and glycopeptides) in patients with septic shock may be altered by changes in the permeability of the microvascular endothelium and consequent alterations in extracellular body water. This may lead to lower than expected plasma concentrations during the first day of therapy resulting in sub-optimal achievement of antibiotic levels [271].

In the setting of alterations in the volume of distribution, loading doses and/or a higher overall total daily dose of beta-lactams, aminoglycosides, or glycopeptides are often required to maximize the pharmodynamics ensuring optimal drug exposure to the infection site in patients with severe sepsis or septic shock [271].

Tissue penetration is also an important aspect because high concentrations at the site of infection can potentially overcome “resistance”. Albumin concentrations are crucial for highly protein-bound drugs [271].

When drains have been inserted, large drainage volume outputs may also affect antibiotic concentration, and may need to be accounted for when considering dosage and frequency of administration.

Once an appropriate initial loading dose is achieved, the antimicrobial regimen should be reassessed, at least daily, because pathophysiological changes may significantly affect drug availability in the critically ill patients. Lower than standard dosages of renally excreted drugs must be administered in the presence of impaired renal function, while higher than standard dosages of renally excreted drugs may be needed for optimal activity in patients with glomerular hyperfiltration [271]. It should be noted that in critically ill patients, plasma creatinine is an unreliable marker of renal function. The phenomenon of “augmented renal clearance” (creatinine clearance >130 ml/min/1.73 m^2^) causes subtherapeutic concentrations. This phenomenon has high prevalence among critically ill patients [278].

### The value of intra-operative specimens

*Obtaining microbiological cultures from blood or fluid/tissue allows:*

- *to expand antimicrobial regimen if the initial choice is too narrow; and*
- *to perform a de-escalation is the empirical regimen is too broad.*

*They should be always performed in patients with healthcare-associated infections or with community-acquired infections at risk for resistant pathogens.*

*When a microorganism is identified in clinical cultures, antimicrobial susceptibility testing (AST) should always be performed and reported to guide antibiotic therapy.*

A lack of impact on patient outcomes by bacteriological cultures has been documented in patients with community-acquired IAI, especially in appendicitis [279, 280]. However, this observation may not address the issues surrounding the threats of antibiotic resistance. The results of microbiological testing may have great importance for the choice of therapeutic strategy of every patient, in particular in the adaptation of targeted antimicrobial treatment. While the yield of blood cultures may be relatively low in patients with IAI, clinicians should not miss an easy opportunity to establish the microbiologic etiology by obtaining two sets of blood cultures prior to starting antibiotics, particularly in patients admitted to the hospital in critically ill conditions. Fluid and/or tissue culture from the site of infection should be collected, particularly in the presence of an abscess. Sufficient fluid volume (usually at least 1 mL of fluid or tissue) must be collected, and then transported to the microbiology laboratory using a transport system that properly handles and preserves the samples to avoid damage or compromise their integrity.

Obtaining microbiological results from blood or fluid/tissue culture from the site of infection has two advantages: a) it provides an opportunity to expand antimicrobial regimen if the initial choice was too narrow, and, b) it also allows de-escalation of antimicrobial therapy if the empirical regimen was too broad. When a microorganism is identified in clinical cultures, antimicrobial susceptibility testing (AST) should always be performed and reported to guide antibiotic therapy. AST measures the ability of a specific organism to grow in the presence of a particular drug using guidelines established by either the Clinical or Laboratory Standards Institute (CLSI) in United States or the European Committee for Antimicrobial Susceptibility Testing (EUCAST) in Europe. By the end of 2012, in several European Countries, CLSI guidelines had been replaced by EUCAST [281]. In vitro susceptibility results are correlated with the clinical success or failure of an antibiotic against a particular organism. Data are reported in the form of MIC, which is the lowest concentration of an antibiotic that inhibits visible growth of a microorganism. The numerical MIC number, expressed as micrograms/ml, is usually reported by microbiology laboratories as a categorical guide for clinicians, ie as “susceptible”, “resistant”, or “intermediate”, according to CLSI and EUCAST criteria. In general, EUCAST supports lower, more stringent, resistance MIC breakpoints than CLSI, in particular for Gram-negative bacteria [282]. However, in only a few cases have these differences been translated into major interpretive category discrepancies [283]. Both CLSI and EUCAST periodically update their recommendations concerning the interpretation of in vitro AST [284]. Recently, both CLSI and EUCAST published new AST guidelines, but some differences in terms of the categorization of ESBLs still remain in the EUCAST guidelines [285]. In general, it may be a wise practice to communicate directly with the microbiology laboratory when antimicrobial susceptibility patterns appear unusual.

### Antimicrobial duration

*In patients with uncomplicated IAI, and where the source of infection is treated definitively, post-operative antimicrobial therapy is not necessary.*

*In patients with complicated IAI undergoing an adequate source-control procedure, post-operative therapy should be shortened as much as possible after the resolution of physiological abnormalities.*

In the event of uncomplicated IAIs, the infection involves a single organ and does not extend to the peritoneum. When the source of infection is treated effectively by surgical excision, post-operative antimicrobial therapy is not necessary, as demonstrated in managing uncomplicated acute appendicitis or cholecystitis [177, 286–288]. In complicated IAI, the infectious process extends beyond the organ, causing either localized or diffuse peritonitis (examples include: perforated appendicitis, perforated peptic ulcer, perforated diverticulitis, and post-operative anastomotic leaks) [289, 290]. Treatment of patients with complicated IAI generally involves both source control and antimicrobial therapy. Antibiotics to treat IAI with antimicrobials can prevent local and hematogenous spread and may reduce late complications.

The optimal duration of antibiotic therapy for cIAIs is debated. Guidelines by Surgical Infection Society (SIS) and Infectious Diseases Society of America (IDSA), published in 2010 [171], recommended a treatment course of 4 to 7 days, depending on the clinical response. French guidelines also recommended 5 to 7 days of treatment [178]. The World Society of Emergency Surgery (WSES) [174] recommended shortened antibiotic therapy in those patients demonstrating a positive response to treatment, without signs of persistent leukocytosis or fever. The recent prospective trial by Sawyer et al. demonstrated that in patients with complicated IAI undergoing an adequate source-control procedure, the outcomes after approximately 4 days fixed-duration antibiotic therapy were similar to those after a longer course of antibiotics that extended until after the resolution of physiological abnormalities [291]. Finally, protracted antibiotic administration may not be safe; for IAI in which a duration of therapy >7 days was prescribed, an association with increased risk of subsequent extra-abdominal infections and increased mortality, was recently observed [292].

Duration of therapy should be shortened as much as possible unless there are special circumstances that require prolonging antimicrobial therapy such as immunosuppression, or ongoing infections. Oral antimicrobials, can substitute IV agents as soon as the patient is tolerating an oral diet so as to minimize the adverse effects which are associated with intravenous access devices. Where possible, conversion to oral antimicrobial agents having high oral bioavailability (e.g. fluoroquinolones) should be considered. Patients who have signs of sepsis beyond 5 to 7 days of treatment warrant aggressive diagnostic investigation to determine if an ongoing uncontrolled source of infection or antimicrobial treatment failure is present. In the management of critically ill patients with sepsis and septic shock clinical signs and symptoms as well as inflammatory response markers such as procalcitonin, although debatable, may assist in guiding antibiotic treatment [293].

Recently, a systematic review of preclinical and clinical studies of mediators in intra-abdominal sepsis/injury was published [294].

Persistently high PCT in plasma was associated with infection or with a significant increase in mortality in patients with sepsis [295, 296].

Therefore, PCT has been used as a guide for interventions or antibiotic therapy for patients with abdominal sepsis [297]. However, other studies have not consistently confirmed PCT as an accurate marker for sepsis or to predict patient’s response to the initial treatment [298].

## Conclusions

An optimal antimicrobial approach to treating IAI involves a delicate balance between the optimization of empiric therapy, which improves clinical outcomes, and the reduction of excessive antimicrobial use, which increases the rate of emergence of antimicrobial-resistant strains. Increasing resistance rates among Gram-negative pathogens that are responsible for serious nosocomial infections, including ESBL *Enterobacteriaceae,* MDR *P. aeruginosa* and carbapenem-resistant *Enterobacteriaceae* is a consequence of increasing acquisition of carbapenemase genes worldwide [299]. These organisms represent an emerging threat due to the limited availability of viable therapeutic options. This complicates the choice of the most appropriate empiric treatment for patients with IAI. The clinical challenge remains to find the balance between ensuring that each individual patient is appropriately covered for the most likely pathogens of their IAI, while avoiding the use of overtly broad-spectrum antimicrobials in order to preserve them for future use. The appropriateness and need for antimicrobial treatment should be re-assessed daily. Treatment duration as short as 4 days may be sufficient for a vast majority of patients suffering from complicated IAIs, when coupled with effective source control.

Although most clinicians are aware of the problem of antimicrobial resistance, most underestimate its importance; judicious antimicrobial management decisions is an integral part of responsible medication prescribing behavior.

In Appendix recommendations for appropriate therapy in patients with intra-abdominal infections are reported.

## Abbreviations

AMR, antimicrobial resistance; IAIs, intra-abdominal infections; CDI, *C. difficile* infection; ESBL, extended-spectrum beta-lactamases; KPC, *K. pneumoniae* carbapenemase; MDRO, multidrug resistant organism; CA-IAIs, community-acquired intra-abdominal infections; HCA-IAIs, healthcare-associated intra-abdominal infections

## Appendix

### Recommendations for appropriate therapy in patients with intra-abdominal infections

- Antimicrobials should be used after a treatable IAI has been recognized or if there is a high degree of suspicion of an infection.
- Patient factors, the nature of the infection and disease, and the environment all affect appropriate planning of antimicrobial therapy.
- Empiric antimicrobial therapy should be started in patients with IAI.
- Knowledge of local rates of resistance is an essential component in the determination of the empiric antimicrobial regimen for IAI.
- For patients with community-acquired IAI, empiric agents with a narrower spectrum of activity are sufficient.
- For patients with hospital-acquired IAI, antimicrobial regimens with broader spectrum of activity are preferred.
- Targeted antimicrobial therapy regimens are appropriate when culture and antimicrobial susceptibility test results are available.
- In uncomplicated IAI, post-operative therapy is not usually necessary following source control.
- In complicated IAIs, antimicrobial therapy is usually continued after source control.
- The antimicrobial therapy should be shortened in patients demonstrating a positive response to treatment.
- Patients having signs of sepsis beyond 5 to 7 days of antibiotic treatment should undergo aggressive diagnostic investigation to determine ongoing uncontrolled sources of infection, or antimicrobial treatment failure.
- Where possible, conversion to oral antimicrobial agents with high oral bioavailability should be considered to minimize the adverse effects associated with intravenous access devices.
- Sufficient knowledge of the general principles of antimicrobial therapy is necessary for clinicians treating intra-abdominal infections; this may minimize treatment failures, and minimize the development of antimicrobial resistance.
