# Erratum to: Antimicrobials: a global alliance for optimizing their rational use in intra-abdominal infections (AGORA)

**DOI:** 10.1186/s13017-017-0147-0

**Published:** 2017-08-02

**Authors:** M. Sartelli, D. G. Weber, E. Ruppé, M. Bassetti, B. J. Wright, L. Ansaloni, F. Catena, F. Coccolini, F. M. Abu-Zidan, R. Coimbra, E. E. Moore, F. A. Moore, R. V. Maier, J. J. De Waele, A. W. Kirkpatrick, E. A. Griffiths, C. Eckmann, A. J. Brink, J. E. Mazuski, A. K. May, R. G. Sawyer, D. Mertz, P. Montravers, A. Kumar, J. A. Roberts, J. L. Vincent, R. R. Watkins, W. Lowman, B. Spellberg, I. J. Abbott, A. K. Adesunkanmi, S. Al-Dahir, M. N. Al-Hasan, F. Agresta, A. A. Althani, S. Ansari, R. Ansumana, G. Augustin, M. Bala, Z. J. Balogh, O. Baraket, A. Bhangu, M. A. Beltrán, M. Bernhard, W. L. Biffl, M. A. Boermeester, S. M. Brecher, J. R. Cherry-Bukowiec, O. R. Buyne, M. A. Cainzos, K. A Cairns, A. Camacho-Ortiz, S. J. Chandy, A. Che Jusoh, A. Chichom-Mefire, C. Colijn, F. Corcione, Y. Cui, D. Curcio, S. Delibegovic, Z. Demetrashvili, B. De Simone, S. Dhingra, J. J. Diaz, I. Di Carlo, A. Dillip, S. Di Saverio, M. P. Doyle, G. Dorj, A. Dogjani, H. Dupont, S. R. Eachempati, M. A. Enani, V. N. Egiev, M. M. Elmangory, P. Ferrada, J. R. Fitchett, G. P. Fraga, N. Guessennd, H. Giamarellou, W. Ghnnam, G. Gkiokas, S. R. Goldberg, C. A. Gomes, H. Gomi, M. Guzmán-Blanco, M. Haque, S. Hansen, A. Hecker, W. R. Heizmann, T. Herzog, A. M. Hodonou, S. K. Hong, R. Kafka-Ritsch, L. J. Kaplan, G. Kapoor, A. Karamarkovic, M. G. Kees, J. Kenig, R. Kiguba, P. K. Kim, Y. Kluger, V. Khokha, K. Koike, K. Y. Kok, V. Kong, M. C. Knox, K. Inaba, A. Isik, K. Iskandar, R. R. Ivatury, M. Labbate, F. M. Labricciosa, P. F. Laterre, R. Latifi, J. G. Lee, Y. R. Lee, M. Leone, A. Leppaniemi, Y. Li, S. Y. Liang, T. Loho, M. Maegele, S. Malama, H. E. Marei, I. Martin-Loeches, S. Marwah, A. Massele, M. McFarlane, R. B. Melo, I. Negoi, D. P. Nicolau, C. E. Nord, R. Ofori-Asenso, A. H. Omari, C. A. Ordonez, M. Ouadii, G. A. Pereira Júnior, D. Piazza, G. Pupelis, T. M. Rawson, M. Rems, S. Rizoli, C. Rocha, B. Sakakushev, M. Sanchez-Garcia, N. Sato, H. A. Segovia Lohse, G. Sganga, B. Siribumrungwong, V. G. Shelat, K. Soreide, R. Soto, P. Talving, J. V. Tilsed, J. F. Timsit, G. Trueba, N. T. Trung, J. Ulrych, H. van Goor, A. Vereczkei, R. S. Vohra, I. Wani, W. Uhl, Y. Xiao, K. C. Yuan, S. K. Zachariah, J. R. Zahar, T. L. Zakrison, A. Corcione, R. M. Melotti, C. Viscoli, P. Viale

**Affiliations:** 1 Department of Surgery, Macerata Hospital, Via Santa Lucia 2, 62100 Macerata, Italy; 2 Department of Trauma Surgery, Royal Perth Hospital, Perth, Australia; 3 Genomic Research Laboratory, Geneva University Hospitals, Geneva, Switzerland; 4 Infectious Diseases Division, Santa Maria Misericordia University Hospital, Udine, Italy; 5 Department of Emergency Medicine and Surgery, Stony Brook University School of Medicine, Stony Brook, NY USA; 6 General Surgery Department, Papa Giovanni XXIII Hospital, Bergamo, Italy; 7 Department of General, Maggiore Hospital, Parma, Italy; 8 Department of Surgery, “Infermi” Hospital, Rimini, Italy; 9 Department of Surgery, College of Medicine and Health Sciences, UAE University, Al-Ain, United Arab Emirates; 10 Department of Surgery, UC San Diego Medical Center, San Diego, USA; 11 Department of Surgery, University of Colorado, Denver Health Medical Center, Denver, CO USA; 12 Department of Surgery, Division of Acute Care Surgery, and Center for Sepsis and Critical Illness Research, University of Florida College of Medicine, Gainesville, FL USA; 13 Department of Surgery, University of Washington, Seattle, WA USA; 14 Department of Critical Care Medicine, Ghent University Hospital, Ghent, Belgium; 15 General, Acute Care, and Trauma Surgery, Foothills Medical Centre, Calgary, AB Canada; 16 General and Upper GI Surgery, Queen Elizabeth Hospital, Birmingham, UK; 17 Department of General, Visceral, and Thoracic Surgery, Klinikum Peine, Academic Hospital of Medical University Hannover, Peine, Germany; 18 Department of Clinical microbiology, Ampath National Laboratory Services, Milpark Hospital, Johannesburg, South Africa; 19 Department of Surgery, School of Medicine, Washington University in Saint Louis, Saint Louis, MO USA; 20 Departments of Surgery and Anesthesiology, Division of Trauma and Surgical Critical Care, Vanderbilt University Medical Center, Nashville, TN USA; 21 Department of Surgery, University of Virginia Health System, Charlottesville, VA USA; 22 Departments of Medicine, Clinical Epidemiology and Biostatistics, and Pathology and Molecular Medicine, McMaster University, Hamilton, ON Canada; 23 Département d’Anesthésie-Réanimation, CHU Bichat Claude-Bernard-HUPNVS, Assistance Publique-Hôpitaux de Paris, University Denis Diderot, Paris, France; 24 Section of Critical Care Medicine and Section of Infectious Diseases, Department of Medicine, Medical Microbiology and Pharmacology/Therapeutics, University of Manitoba, Winnipeg, MB Canada; 25 Australia Pharmacy Department, Royal Brisbane and Womens’ Hospital, Burns, Trauma, and Critical Care Research Centre, Australia School of Pharmacy, The University of Queensland, QLD, Brisbane, Australia; 26 Department of Intensive Care, Erasme Hospital, Université libre de Bruxelles, Brussels, Belgium; 27 Department of Internal Medicine, Division of Infectious Diseases, Akron General Medical Center, Northeast Ohio Medical University, Akron, OH USA; 28 Clinical Microbiology and Infectious Diseases, School of Pathology, Faculty of Health Sciences, University of the Witwatersrand, Johannesburg, South Africa; 29 Division of Infectious Diseases, Los Angeles County-University of Southern California (USC) Medical Center, Keck School of Medicine at USC, Los Angeles, CA USA; 30 Department of Infectious Diseases, Alfred Hospital, Melbourne, VIC Australia; 31 Department of Surgery, College of Health Sciences, Obafemi Awolowo University, Ile-Ife, Nigeria; 32 Division of Clinical and Administrative Sciences, College of Pharmacy, Xavier University of Louisiana, New Orleans, LA USA; 33 Department of Medicine, Division of Infectious Diseases, University of South Carolina School of Medicine, Columbia, SC USA; 34 General Surgery, ULSS19 del Veneto, Adria Hospital, Adria, RO Italy; 35 Biomedical Research Center, Qatar University, Doha, Qatar; 36 Department of Microbiology, Chitwan Medical College, and Department of Environmental and Preventive Medicine, Oita University, Oita, Japan; 37 Centre for Neglected Tropical Diseases, Liverpool School of Tropical Medicine, University of Liverpool, and Mercy Hospital Research Laboratory, Njala University, Bo, Sierra Leone; 38 Department of Surgery, University Hospital Center, Zagreb, Croatia; 39 Trauma and Acute Care Surgery Unit, Hadassah Hebrew University Medical Center, Jerusalem, Israel; 40 Department of Traumatology, John Hunter Hospital and University of Newcastle, Newcastle, NSW Australia; 41 Department of Surgery, Bizerte Hospital, Bizerte, Tunisia; 42 Academic Department of Surgery, Queen Elizabeth Hospital, Birmingham, UK; 43 Department of General Surgery, Hospital San Juan de Dios de La Serena, La Serena, Chile; 44 Emergency Department, University of Leipzig, Leipzig, Germany; 45 Department of Surgery, University of Colorado, Denver, CO USA; 46 Department of Surgery, Academic Medical Centre, Amsterdam, The Netherlands; 47 Department of Pathology and Laboratory Medicine, VA Boston HealthCare System, and Department of Pathology and Laboratory Medicine, Boston University School of Medicine, Boston, MA USA; 48 Division of Acute Care Surgery, Department of Surgery, University of Michigan, Ann Arbor, MI USA; 49 Department of Surgery, Radboud University Nijmegen Medical Center, Nijmegen, The Netherlands; 50 Department of Surgery, Hospital Clínico Universitario, Santiago de Compostela, Spain; 51 Pharmacy Department, Alfred Health, Melbourne, VIC Australia; 52 Hospital Epidemiology and Infectious Diseases, Hospital Universitario Dr Jose Eleuterio Gonzalez, Monterrey, Mexico; 53 Department of Pharmacology, Pushpagiri Institute of Medical Sciences and Research Centre, Thiruvalla, Kerala India; 54 Department of General Surgery, Kuala Krai Hospital, Kuala Krai, Kelantan Malaysia; 55 Department of Surgery and Obstetrics/Gynaecology, Regional Hospital, Limbe, Cameroon; 56 Department of Mathematics, Imperial College London, London, UK; 57 Department of Laparoscopic and Robotic Surgery, Colli-Monaldi Hospital, Naples, Italy; 58 Department of Surgery, Tianjin Nankai Hospital, Nankai Clinical School of Medicine, Tianjin Medical University, Tianjin, China; 59 Infectología Institucional SRL, Hospital Municipal Chivilcoy, Buenos Aires, Argentina; 60 Department of Surgery, University Clinical Center of Tuzla, Tuzla, Bosnia and Herzegovina; 61 Department General Surgery, Kipshidze Central University Hospital, Tbilisi, Georgia; 62 Department of Surgery, Quatre Villes Hospital, St Cloud, France; 63 School of Pharmacy, Faculty of Medical Sciences, The University of the West Indies, St. Augustine, Eric Williams Medical Sciences Complex, Uriah Butler Highway, Champ Fleurs, Trinidad and Tobago; 64 Division of Acute Care Surgery, Program in Trauma, R Adams Cowley Shock Trauma Center, University of Maryland, Baltimore, MD USA; 65 Department of Surgical Sciences, Cannizzaro Hospital, University of Catania, Catania, Italy; 66 Ifakara Health Institute, Dar es Salaam, Tanzania; 67 Department of Surgery, Maggiore Hospital, Bologna, Italy; 68 Center for Food Safety, Department of Food Science and Technology, University of Georgia, Griffin, GA USA; 69 School of Pharmacy and Biomedicine, Mongolian National University of Medical Sciences, Ulaanbaatar, Mongolia; 70 Department of Surgery, University Hospital of Trauma, Tirana, Albania; 71 Département d’Anesthésie-Réanimation, CHU Amiens-Picardie, and INSERM U1088, Université de Picardie Jules Verne, Amiens, France; 72 Department of Surgery, Division of Burn, Critical Care, and Trauma Surgery (K.P.S., S.R.E.), Weill Cornell Medical College/New York-Presbyterian Hospital, New York, USA; 73 Department of Medicine, Infectious Disease Division, King Fahad Medical City, Riyadh, Saudi Arabia; 74 Department of Surgery, Pirogov Russian National Research Medical University, Moscow, Russian Federation; 75 Sudan National Public Health Laboratory, Federal Ministry of Health, Khartoum, Sudan; 76 Department of Surgery, Virginia Commonwealth University, Richmond, VA USA; 77 Department of Global Health and Population, Harvard T.H. Chan School of Public Health, Boston, MA USA; 78 Division of Trauma Surgery, Department of Surgery, School of Medical Sciences, University of Campinas (Unicamp), Campinas, SP Brazil; 79 Institut Pasteur, Abidjan, Ivory Coast; 80 6th Department of Internal Medicine, Hygeia General Hospital, Athens, Greece; 81 Department of General Surgery, Mansoura Faculty of Medicine, Mansoura University, Mansoura, Egypt; 82 2nd Department of Surgery, Aretaieion University Hospital, National and Kapodistrian University of Athens, Athens, Greece; 83 Department of Surgery, Hospital Universitário Terezinha de Jesus, Faculdade de Ciências Médicas e da Saúde de Juiz de Fora, Juiz de Fora, Brazil; 84 Center for Global Health, Mito Kyodo General Hospital, University of Tsukuba, Mito, Ibaraki Japan; 85 Hospital Privado Centro Médico de Caracas and Hospital Vargas de Caracas, Caracas, Venezuela; 86 Unit of Pharmacology, Faculty of Medicine and Defense Health, National Defence University of Malaysia, Kuala Lumpur, Malaysia; 87 Institute of Hygiene, Charité-Universitätsmedizin Berlin, Hindenburgdamm 27, 12203 Berlin, Germany; 88 Department of General and Thoracic Surgery, University Hospital Giessen, Giessen, Germany; 89 Orgamed Consulting, Bad Griesbach, Germany; 90 Department of Surgery, St. Josef Hospital, Ruhr University Bochum, Bochum, Germany; 91 Department of Surgery, Faculté de médecine, Université de Parakou, BP 123, Parakou, Bénin; 92 Division of Trauma and Surgical Critical Care, Department of Surgery, Asan Medical Center, University of Ulsan College of Medicine, Seoul, Republic of Korea; 93 Department of Visceral, Transplant and Thoracic Surgery, Innsbruck Medical University, Innsbruck, Austria; 94 Department of Surgery Philadelphia VA Medical Center, Perelman School of Medicine, University of Pennsylvania, Philadelphia, PA USA; 95 Department of Microbiology, Gandhi Medical College, Bhopal, India; 96 Clinic for Emergency Surgery, Medical Faculty University of Belgrade, Belgrade, Serbia; 97 Department of Anesthesiology and Intensive Care, Charité Universitätsmedizin Berlin, Campus Benjamin Franklin, Berlin, Germany; 98 3rd Department of General Surgery, Jagiellonian University Medical College, Krakow, Poland; 99 Department of Pharmacology and Therapeutics, College of Health Sciences, Makerere University, Kampala, Uganda; 100 Department of Surgery, Albert Einstein College of Medicine and Jacobi Medical Center, Bronx, NY USA; 101 Department of General Surgery, Division of Surgery, Rambam Health Care Campus, Haifa, Israel; 102 Department of Emergency Surgery, City Hospital, Mozyr, Belarus; 103 Department of Primary Care and Emergency Medicine, Kyoto University Graduate School of Medicine, Kyoto, Japan; 104 Department of Surgery, The Brunei Cancer Centre, Jerudong Park, Brunei; 105 Department of Surgery, Edendale Hospital, Pietermaritzburg, South Africa; 106 School of Medicine, Western Sydney University, Campbelltown, NSW Australia; 107 Division of Acute Care Surgery and Surgical Critical Care, Department of Surgery, Los Angeles County and University of Southern California Medical Center, University of Southern California, Los Angeles, CA USA; 108 Department of General Surgery, Erzincan University, Faculty of Medicine, Erzincan, Turkey; 109 Department of Pharmacy, Lebanese International University, Beirut, Lebanon; 110 School of Life Science and The ithree Institute, University of Technology, Sydney, NSW Australia; 111 Department of Biomedical Sciences and Public Health, Unit of Hygiene, Preventive Medicine and Public Health, UNIVMP, Ancona, Italy; 112 Department of Critical Care Medicine, Cliniques Universitaires Saint Luc, Université Catholique de Louvain (UCL), Brussels, Belgium; 113 Department of Surgery, Division of Trauma, University of Arizona, Tucson, AZ USA; 114 Department of Surgery, Yonsei University College of Medicine, Seoul, South Korea; 115 Texas Tech University Health Sciences Center School of Pharmacy, Abilene, TX USA; 116 Department of Anaesthesiology and Critical Care, Hôpital Nord, Assistance Publique-Hôpitaux de Marseille, Aix Marseille Université, Marseille, France; 117 Abdominal Center, University Hospital Meilahti, Helsinki, Finland; 118 Department of Surgery, Inling Hospital, Nanjing University School of Medicine, Nanjing, China; 119 Division of Infectious Diseases, Division of Emergency Medicine, Washington University School of Medicine, St. Louis, MO USA; 120 Division of Infectious Diseases, Department of Clinical Pathology, Faculty of Medicine, University of Indonesia, Cipto Mangunkusumo General Hospital, Jakarta, Indonesia; 121 Department for Traumatology and Orthopedic Surgery, Cologne Merheim Medical Center (CMMC), University of Witten/Herdecke (UW/H), Cologne, Germany; 122 Health Research Program, Institute of Economic and Social Research, University of Zambia, Lusaka, Zambia; 123 Multidisciplinary Intensive Care Research Organization (MICRO), Wellcome Trust-HRB Clinical Research, Department of Clinical Medicine, Trinity Centre for Health Sciences, St James’ University Hospital, Dublin, Ireland; 124 Department of Surgery, Post-Graduate Institute of Medical Sciences, Rohtak, India; 125 Department of Clinical Pharmacology, School of Medicine, University of Botswana, Gaborone, Botswana; 126 Department of Surgery, Radiology, University Hospital of the West Indies, Kingston, Jamaica; 127 General Surgery Department, Centro Hospitalar de São João, Porto, Portugal; 128 Department of Surgery, Emergency Hospital of Bucharest, Bucharest, Romania; 129 Center of Anti-Infective Research and Development, Hartford, CT USA; 130 Department of Laboratory Medicine, Division of Clinical Microbiology, Karolinska University Hospital Huddinge, Stockholm, Sweden; 131 Research Unit, Health Policy Consult, Weija, Accra, Ghana; 132 Department of Surgery, King Abdullah University Hospital, Irbid, Jordan; 133 Department of Surgery and Critical Care, Universidad del Valle, Fundación Valle del Lili, Cali, Colombia; 134 Department of Surgery, Hassan II University Hospital, Medical School of Fez, Sidi Mohamed Benabdellah University, Fez, Morocco; 135 Division of Emergency and Trauma Surgery, Ribeirão Preto Medical School, Ribeirão Preto, Brazil; 136 Division of Surgery, Vittorio Emanuele Hospital, Catania, Italy; 137 Department of General and Emergency Surgery, Riga East University Hospital ‘Gailezers’, Riga, Latvia; 138 National Institute for Health Research, Health Protection Research Unit in Healthcare Associated Infections and Antimicrobial Resistance, Imperial College London, Hammersmith Campus, London, UK; 139 Department of General Surgery, Jesenice General Hospital, Jesenice, Slovenia; 140 Trauma and Acute Care Service, St Michael’s Hospital, University of Toronto, Toronto, Canada; 141 U.S. Naval Medical Research Unit N° 6, Callao, Peru; 142 General Surgery Department, Medical University, University Hospital St George, Plovdiv, Bulgaria; 143 Intensive Care Department, Hospital Clínico San Carlos, Madrid, Spain; 144 II Cátedra de Clínica Quirúrgica, Hospital de Clínicas, Universidad Nacional de Asunción, San Lorenzo, Paraguay; 145 Department of Surgery, Catholic University of Sacred Heart, Policlinico A Gemelli, Rome, Italy; 146 Department of Surgery, Faculty of Medicine, Thammasat University Hospital, Thammasat University, Pathum Thani, Thailand; 147 Department of General Surgery, Tan Tock Seng Hospital, Tan Tock Seng, Singapore, Singapore; 148 Department of Gastrointestinal Surgery, Stavanger University Hospital, Stavanger, Department of Clinical Medicine, University of Bergen, Bergen, Norway; 149 Department of Emergency Surgery and Critical Care, Centro Medico Imbanaco, Cali, Colombia; 150 Department of Surgery, North Estonia Medical Center, Tallinn, Estonia; 151 Surgery Health Care Group, Hull and East Yorkshire Hospitals NHS Trust, Hull, UK; 152 APHP medical and infectious diseases ICU, Bichat Hospital, Paris, France; 153 Institute of Microbiology, Biological and Environmental Sciences College, University San Francisco de Quito, Quito, Ecuador; 154 Department of Molecular Biology, Tran Hung Dao Hospital, No 1, Tran Hung Dao Street, Hai Ba Trung Dist, Hanoi, Vietnam; 155 1st Department of Surgery - Department of Abdominal, Thoracic Surgery and Traumatology, General University Hospital, Prague, Czech Republic; 156 Department of Surgery, Medical School University of Pécs, Pécs, Hungary; 157 Nottingham Oesophago-Gastric Unit, Nottingham University Hospitals, Nottingham, UK; 158 Department of Surgery, Sheri-Kashmir Institute of Medical Sciences, Srinagar, India; 159 State Key Laboratory for Diagnosis and Treatment of Infectious Diseases, The First Affilliated Hospital, Zhejiang University, Zhejiang, China; 160 Trauma and Emergency Surgery Department, Chang Gung Memorial Hospital, Taoyuan City, Taiwan; 161 Department of Surgery, MOSC Medical College Kolenchery, Cochin, India; 162 Infection Control Unit, Angers University, CHU d’Angers, Angers, France; 163 Division of Trauma and Surgical Critical Care, DeWitt Daughtry Family Department of Surgry, University of Miami, Miami, FL USA; 164 Anesthesia and Intensive Care Unit, AORN dei Colli Vincenzo Monaldi Hospital, Naples, Italy; 165 Anesthesiology and Intensive Care Unit, Sant’Orsola University Hospital, Bologna, Italy; 166 Infectious Diseases Unit, University of Genoa (DISSAL) and IRCCS San Martino-IST, Genoa, Italy; 167 Infectious Diseases Unit, Department of Medical and Surgical Sciences, Sant’ Orsola Hospital, University of Bologna, Bologna, Italy

## Erratum

The original article [1] contains an error whereby a co-author, Boris Sakakushev has their family name spelt incorrectly as ‘Sakakhushev’.

The authors would therefore like it known that the correct spelling of the family name is ‘Sakakushev’.

## References

[1] Sartelli M (2016). Antimicrobials: a global alliance for optimizing their rational use in intra-abdominal infections (AGORA). World J Emerg Surg.

